# An Unprecedented Tolerance to Deletion of the Periplasmic Chaperones SurA, Skp, and DegP in the Nosocomial Pathogen Acinetobacter baumannii

**DOI:** 10.1128/jb.00054-22

**Published:** 2022-09-15

**Authors:** Karolin Birkle, Fabian Renschler, Angel Angelov, Gottfried Wilharm, Mirita Franz-Wachtel, Boris Maček, Erwin Bohn, Elena Weber, Jennifer Müller, Lea Friedrich, Monika Schütz

**Affiliations:** a Institut für Medizinische Mikrobiologie und Hygiene, Tübingen, Germany; b NGS Competence Center Tübingen (NCCT), Tübingen, Germany; c Robert Koch-Institute, Wernigerode Branch, Wernigerode, Germany; d Proteome Center Tübingen, Universität Tübingen, Tübingen, Germany; e German Center for Infection Research (DZIF), partner site Tübingen, Tübingen, Germany; University of California San Francisco

**Keywords:** *Acinetobacter baumannii* AB5075, periplasmic chaperones, SurA, OMP biogenesis

## Abstract

The outer membrane (OM) of Gram-negative bacteria efficiently protects from harmful environmental stresses such as antibiotics, disinfectants, or dryness. The main constituents of the OM are integral OM β-barrel proteins (OMPs). In Gram-negative bacteria such as Escherichia coli, Yersinia enterocolitica, and Pseudomonas aeruginosa, the insertion of OMPs depends on a sophisticated biogenesis pathway. This comprises the SecYEG translocon, which enables inner membrane (IM) passage; the chaperones SurA, Skp, and DegP, which facilitate the passage of β-barrel OMPs through the periplasm; and the β-barrel assembly machinery (BAM), which facilitates insertion into the OM. In E. coli, Y. enterocolitica, and P. aeruginosa, the deletion of SurA is particularly detrimental and leads to a loss of OM integrity, sensitization to antibiotic treatment, and reduced virulence. In search of targets that could be exploited to develop compounds that interfere with OM integrity in Acinetobacter baumannii, we employed the multidrug-resistant strain AB5075 to generate single gene knockout strains lacking individual periplasmic chaperones. In contrast to E. coli, Y. enterocolitica, and P. aeruginosa, AB5075 tolerates the lack of SurA, Skp, or DegP with only weak mutant phenotypes. While the double knockout strains Δ*surA*Δ*skp* and Δ*surA*Δ*degP* are conditionally lethal in E. coli, all double deletions were well tolerated by AB5075. Strikingly, even a triple-knockout strain of AB5075, lacking *surA*, *skp*, and *degP*, was viable.

**IMPORTANCE** Acinetobacter baumannii is a major threat to human health due to its ability to persist in the hospital environment, resistance to antibiotic treatment, and ability to deploy multiple and redundant virulence factors. In a rising number of cases, infections with multidrug-resistant A. baumannii end up fatally, because all antibiotic treatment options fail. Thus, novel targets have to be identified and alternative therapeutics have to be developed. The knockout of periplasmic chaperones has previously proven to significantly reduce virulence and even break antibiotic resistance in other Gram-negative pathogens. Our study in A. baumannii demonstrates how variable the importance of the periplasmic chaperones SurA, Skp, and DegP can be and suggests the existence of mechanisms allowing A. baumannii to cope with the lack of the three periplasmic chaperones.

## INTRODUCTION

The outer membrane (OM) of Gram-negative bacteria is a selective protective barrier that efficiently prevents the entry of toxic compounds (1). Furthermore, the OM contributes to the resistance against various environmental stresses: it facilitates the secretion of matrices that enable biofilm formation and of enzymes that hydrolyze or inactivate antibiotics, and it enables the secretion of proteins involved in the formation of capsules. Thereby, the OM dampens the efficacy of disinfectants and antibiotic drugs (2). Furthermore, the OM harbors numerous outer membrane proteins (OMPs), which play important roles in antibiotic resistance, virulence, and immune evasion (1).

OMPs contribute to the active outflow of drugs as components of efflux pumps or mediate direct interaction with host cells and host cell factors. A crucial step in the biogenesis of these OMPs, and of β-barrel OMPs in general, is their stabilization and protection during the passage across the periplasm. Being very hydrophobic in nature, β-barrel OMPs tend to aggregate and misfold in aqueous environments such as the periplasm (3). Therefore, periplasmic chaperones prevent their aggregation and misfolding by keeping them in an unfolded but folding-competent state until the β-barrel assembly machinery (BAM) complex is reached. This multiprotein complex, which is anchored to the OM via the essential β-barrel protein BamA, facilitates the insertion of β-barrel OMPs (4). The decisive role of periplasmic chaperones and especially of SurA in OMP biogenesis and thus OM integrity has been recognized already many years ago (5). Consequently, periplasmic chaperones have been considered potential drug targets in Escherichia coli (6, 7), Yersinia enterocolitica (8), and Pseudomonas aeruginosa (9). Most of our knowledge about periplasmic chaperones is derived from studies in E. coli, in which the most important periplasmic chaperones are SurA, Skp, and DegP (3).

Survival factor A or SurA is the main chaperone for nascent OMPs in E. coli (10, 11) and was originally described as a protein that is essential for cell survival during the stationary phase (12). Later on, its chaperone activity could be demonstrated (5, 13). In E. coli, the lack of SurA leads to distinct phenotypes that resemble those induced by a σ^E^ stress response. These phenotypes include reduced levels of OMPs and increased sensitivity to treatment with detergents like bile salts (5, 10, 13). Moreover, a reduction of virulence upon deletion of SurA has been demonstrated in several species such as uropathogenic E. coli (6), Y. enterocolitica (8), Yersinia pseudotuberculosis (14), as well as in Salmonella enterica (15). In P. aeruginosa, a knockout of SurA even seems to be lethal. Using a conditional knockout mutant instead, we recently demonstrated that in P. aeruginosa SurA is crucial for virulence, resistance against antibiotics, human serum and bile salts, as well as for the OM barrier function of P. aeruginosa (9).

With SurA being the key chaperone at least in E. coli, Y. enterocolitica, and P. aeruginosa, the periplasmic 17-kDa protein, or Skp, appears to play a subsidiary role in shuttling OMPs through the periplasm in most cases (8–10, 16). However, an important role for Skp in the assembly of specific OMPs like LptD has been reported (17). In Neisseria meningitidis, Skp instead of SurA has been identified as the main periplasmic chaperone (18).

DegP, also known as high-temperature requirement A (HtrA), is another important periplasmic chaperone. Besides its chaperone activity, DegP can also act as a protease that degrades misfolded OMPs that have accumulated in the periplasm (10, 19, 20). At low temperatures, the chaperone activity prevails, whereas at elevated temperatures DegP mainly acts as a protease. In line with this observation, DegP seems to be essential for the survival of E. coli at high temperatures (19). A lack of DegP, however, did not induce detectable phenotypes with regard to susceptibility against antibiotics, human serum, and detergents in Y. enterocolitica and P. aeruginosa (8, 9), whereas in E. coli minor defects have been reported (21).

To examine the importance of the single periplasmic chaperones in the transport and stabilization of nascent OMPs and to figure out whether they act redundantly, double knockouts have also been analyzed in E. coli. Concomitant deletion of *surA* and *skp* or of *surA* and *degP* was not tolerated by E. coli (11). Therefore, the existence of two pathways that act in parallel in the transport of nascent OMPs through the periplasm was proposed. Based on this model, SurA as key chaperone represents the default pathway for the majority of OMPs, whereas Skp and DegP play a minor role and operate as a rescue pathway for misfolded OMPs or OMPs that have fallen off the SurA pathway (10, 11). For some OMPs, however, a clear specificity for SurA has been shown, as their OM insertion is massively impaired in the absence of SurA, even in the presence of Skp and DegP (8, 9, 16).

Heading the list of “priority pathogens” of antibiotic-resistant bacteria published by the World Health Organization (WHO), A. baumannii has been recognized as a major threat to health care systems worldwide (22). A. baumannii is a very successful nosocomial pathogen with a great capability to persist in the hospital environment. It can cause a variety of diseases such as bacteremia, meningitis, and wound and skin infections. In intensive care units (ICUs), ventilator-associated pneumonia and bloodstream infections are among the most frequent diseases caused by A. baumannii, and both are associated with high morbidity and lethality (23). Apart from its numerous and often redundantly acting virulence factors making it such a successful pathogen, A. baumannii has high intrinsic resistance. Additionally, further resistance determinants can be acquired extremely well by means of its high natural competence (24). Consequently, the urgency to find alternative therapies to fight infections by resistant strains of A. baumannii has tremendously increased in the last few years. With the identification of the periplasmic chaperone proteins, and especially of SurA as potential drug targets in other bacterial species, we aimed to decipher the role of SurA, Skp, and DegP in OM integrity, antibiotic susceptibility, fitness, and virulence of the nosocomial pathogen A. baumannii. Therefore, single *surA*, *skp*, or *degP* null mutant strains were generated in the multidrug-resistant (MDR) A. baumannii strain AB5075 (25). Surprisingly, the single knockout strains of *surA*, *skp,* or *degP* had only weak mutant phenotypes. Due to these mild defects compared to those we had observed previously in Y. enterocolitica and P. aeruginosa, we created the double knockouts strains Δ*surA*Δ*skp*, Δ*surA*Δ*degP*, and Δ*skp*Δ*degP* and even a triple knockout, lacking all three chaperones. Strikingly, and in contrast to what had been reported before for E. coli (11), the double knockouts were well tolerated by AB507*5*, again causing mostly mild defects. Simultaneous deletion of *surA* and *skp* had the greatest effect on antibiotic susceptibility and virulence and caused a break of resistance against aminoglycosides. Most surprisingly, even the triple-knockout strain was viable. Taken together, we show that A. baumannii behaves distinctly from E. coli, Y. enterocolitica, and P. aeruginosa, thus questioning the suitability of periplasmic chaperones as drug targets in A. baumannii. Moreover, our results suggest the existence of mechanisms that allow A. baumannii to tolerate or compensate for the concurrent lack of the three periplasmic chaperones SurA, Skp, and DegP.

## RESULTS

### Creation of markerless deletions of genes encoding the periplasmic chaperones SurA, Skp, and DegP in AB5075.

To determine the role of periplasmic chaperones in A. baumannii we used a MDR strain, AB5075, which has been sequenced, is well characterized, and is genetically accessible (25, 26). To generate single gene deletion mutants for *surA*, *skp*, and *degP*, we used pVT77 as a markerless genome-editing tool (27). All deletions were verified by PCR (Fig. S1) and by sequencing of the scar regions. First, we analyzed whether the knockout strains have any defects with regard to growth (Fig. S2). None of the single gene knockout mutants displayed a growth defect at either 37°C or 42°C in Luria Bertani (LB) medium. However, the mutant lacking DegP reached a higher maximum optical density at 600 nm (OD_600nm_) value than the other mutants at both temperatures. At 42°C, the OD_600nm_ of all strains started to decline upon reaching the stationary phase.

### Effects on the OM integrity of AB5075.

**FIG 1:**
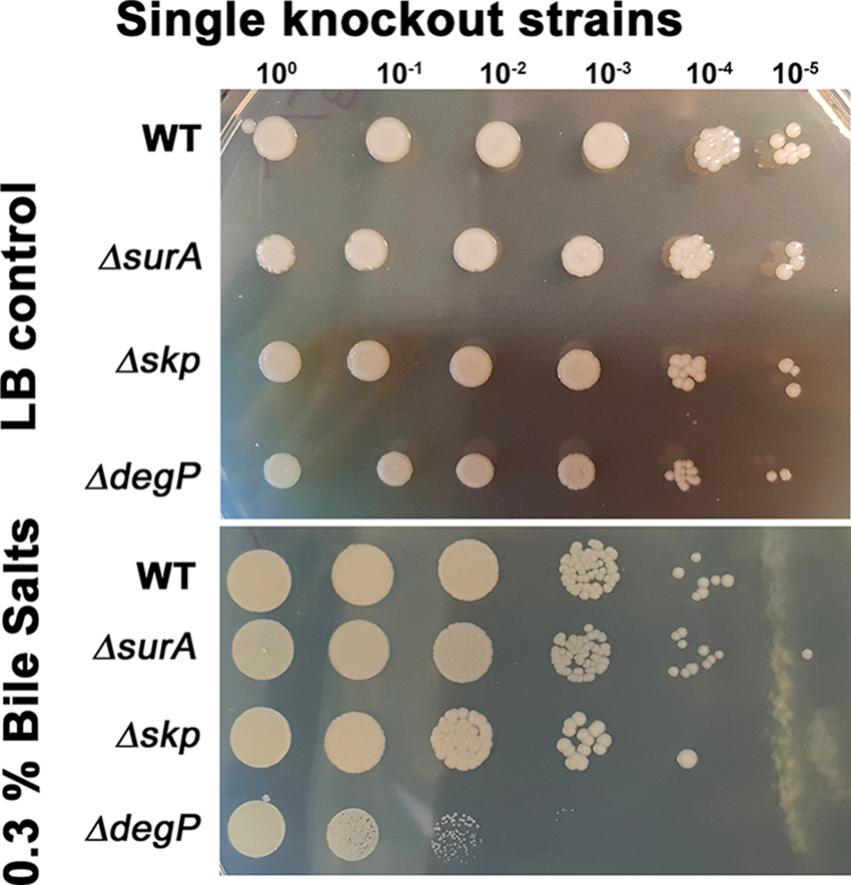
Relevance of the periplasmic chaperones SurA, Skp, and DegP for growth in the presence of bile salts. The growth behavior of the wild type (WT) and the single null strains in the absence and presence of bile salts was compared. The indicated strains were adjusted to 1 × 10^7^ bacteria/ml, serially diluted, and spotted onto LB agar or LB agar containing 0.3% bile salts. At least three experiments were performed. Pictures were taken after an overnight incubation at 37°C (please note: different spot size of the control and treatment group result from different spacing of spots and different spreading behavior of liquid on the plates).

As deletion of SurA has previously been associated with OM integrity defects (5, 8, 9, 13), we tested the susceptibility of the strains toward bile salts. To this end, we spotted serial dilutions of cultures on solid LB, as a control, and on solid LB supplemented with 0.3% bile salts (a condition that barely allowed growth of the wild type). Only the Δ*degP* strain was impaired in growth in the presence of bile salts, whereas the Δ*surA* and the Δ*skp* strains grew like the wild type (Fig. 1). This was in contrast to our previous analyses in Y. enterocolitica and P. aeruginosa (8, 9) in which we detected sensitivity toward bile salts only in the absence of SurA. Therefore, we investigated whether the A. baumannii periplasmic chaperones may differ in relevance and function compared to those of Y. enterocolitica and P. aeruginosa.

### Apparent redundant functions for the periplasmic chaperones SurA, Skp, and DegP in AB5075.

In E. coli, SurA, Skp, and DegP are at least partially redundant. Simultaneous disruption of the chaperoning pathway maintained by SurA, and the alternative pathway dependent on Skp/DegP, results in synthetic phenotypes (10, 11). Therefore, we tested whether the concomitant deletion of two of the periplasmic chaperones might have a similar effect in AB5075. To this end, we generated different combinations of double mutants and succeeded also in generating a triple-knockout strain lacking *surA*, *skp,* and *degP* without any problems. All deletions were verified by PCR, sequencing of the scar region, and RT-PCR (Fig. S3). Moreover, we performed whole-genome sequencing of the parent strain as well as of the double and triple mutants confirming the target deletion of the chaperone encoding genes (data available at the European Nucleotide Archive [ENA], study ID PRJEB40766). Again, we recorded growth curves at 37°C and 42°C (Fig. S4). At 37°C, the Δ*surA*Δ*skp*, Δ*skp*Δ*degP*, and the triple knockout strains grew slower than the wild-type strain and the triple mutant displayed the most pronounced growth defect. At 42°C we saw the strongest phenotypes for the Δ*surA*Δ*degP* and the Δ*surA*Δ*skp*Δ*degP* strain.

### Phenotyping the double and triple periplasmic chaperone mutants of AB5075.

**FIG 2:**
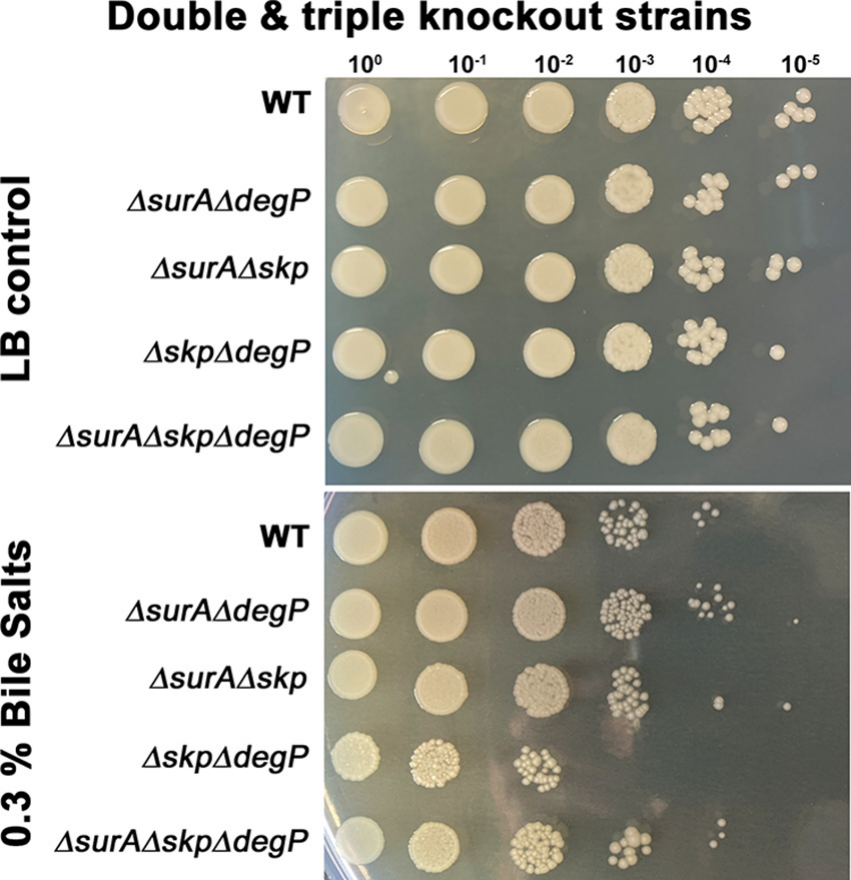
Relevance of the periplasmic chaperones SurA, Skp, and DegP for growth in the presence of detergent. The growth behavior of double and triple knockout strains for the periplasmic chaperones SurA, Skp, and DegP in the absence and presence of bile salts was compared to that of the parent strain AB5075 (WT). The indicated strains were adjusted to 1 × 10^7^ bacteria/ml, serially diluted, and spotted onto LB agar and LB agar containing 0.3% bile salts. The dilution factor is indicated on top of the photograph. At least three experiments were performed. Pictures were taken after an overnight cultivation at 37°C.

**FIG 3:**
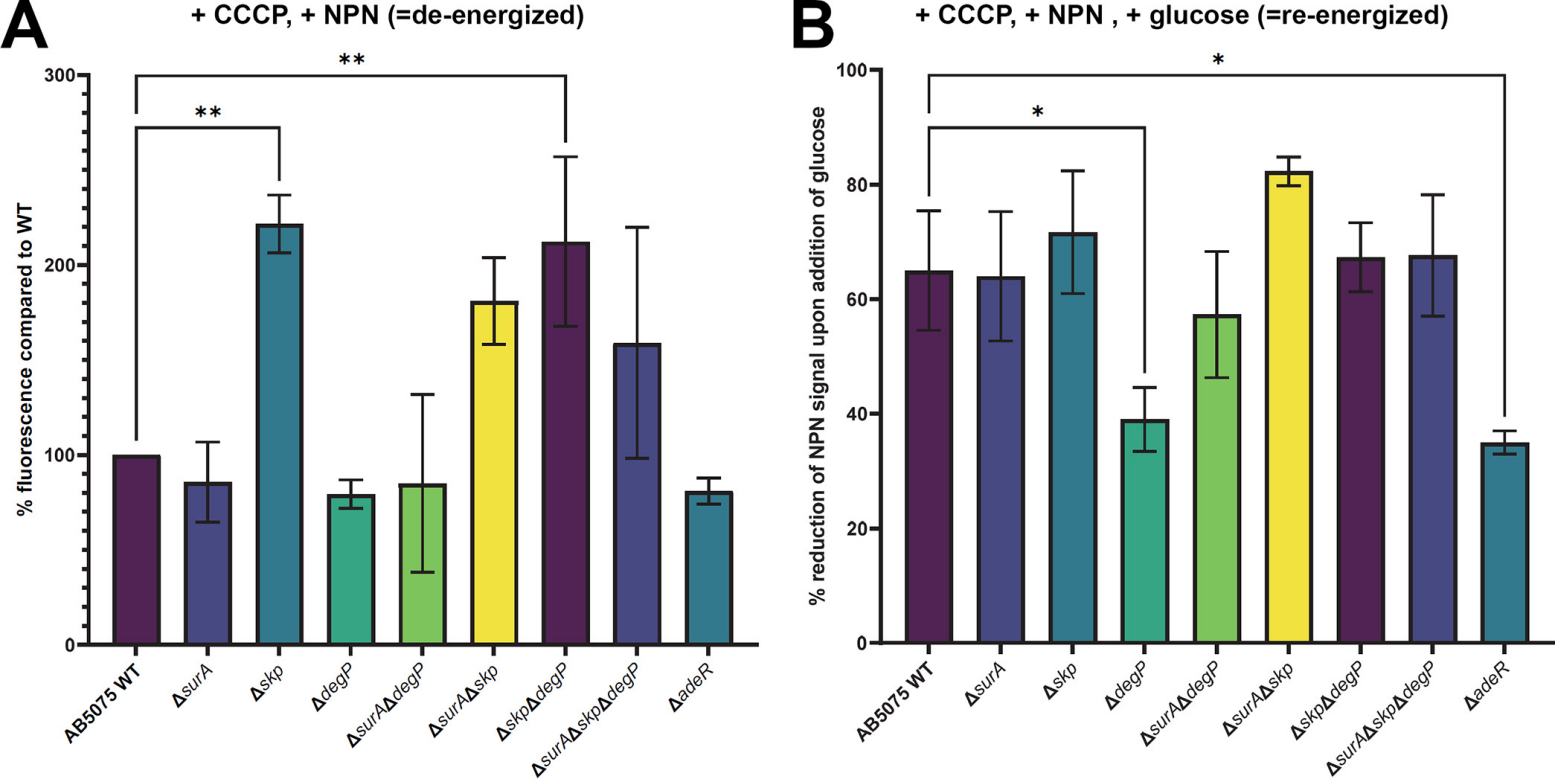
Probing membrane integrity and efflux using 1-*N*-phenylnaphthylamine (NPN). (A) Membrane integrity. Before loading with NPN, cells were deenergized by treatment with CCCP, thereby preventing active expulsion of NPN. The wild-type (WT), single, double, and triple knockout strains, as well as a Δ*adeR* control strain, hampered in expression of the AdeABC efflux pump, were incubated with NPN which emits fluorescence only in hydrophobic environments. Higher fluorescence therefore indicates a reduced OM integrity. (B) NPN efflux. By addition of glucose, cells were reenergized to allow for the active expulsion of NPN. Fluorescence was recorded immediately after injection of glucose. Data show means of three independent experiments. Statistical analysis was performed using one-way ANOVA analysis. Asterisks designate significant differences (**, *P* = 0.001 to 0.01 or *, *P* = 0.01 to 0.05).

**(i) OM integrity and efflux activity.** Our initial hypothesis was that the deletion of the periplasmic chaperones will induce changes in the OM composition. Therefore, we assessed OM integrity, again using a bile salts assay (Fig. 2). Of note, only the strain lacking both Skp and DegP displayed a reduced growth whereas further deletion of SurA rescued the growth defect of the Δ*skp*Δ*degP* mutant strain. To corroborate these interesting but unexpected results, we probed membrane integrity with another assay, i.e., the 1-N-phenylnaphthylamine (NPN)-assay (Fig. 3). NPN is fluorescent only in hydrophobic environments. If the integrity of the OM is compromised, NPN can reach the phospholipid bilayer of the inner OM leaflet more efficiently (28). Higher fluorescence, therefore, indicates a reduced OM integrity. To uncouple NPN uptake and expulsion, we treated the cells with carbonyl cyanide 3-chlorophenylhydrazone (CCCP) before loading them with NPN. This deenergizes the cells and therefore blocks efflux pumps (29, 30). As a control, we used the Δ*adeR* strain, which is hampered in the expression of the AdeABC efflux pump. We recorded the NPN-mediated fluorescence, normalized it to the OD_600nm_ also measured in the wells, and compared it to the wild type (Fig. 3A). Essentially, we found that all strains lacking Skp displayed an increase in the uptake of NPN, being significant in the Δ*skp* and the Δ*skp*Δ*degP* mutant strains. The Δ*surA*, Δ*degP*, Δ*adeR*, and the Δ*surA*Δ*degP* mutant strains, however, did not reveal significant differences in NPN uptake compared to wild type. Next, cells were reenergized by the addition of glucose, and changes in fluorescence were recorded immediately (Fig. 3B). Taken together, we found significantly less reduction of the NPN signal in the Δ*adeR* control strain and to a similar degree in the Δ*degP* mutant strain. This means that the absence of DegP alone is sufficient to impair the efficient removal of NPN from the interior of the cells. These results could possibly explain the phenotype we had observed with the Δ*degP* strain in the bile salts assay as it has been shown previously for other species that efflux pumps can also be involved in the removal of bile salts (31).

**(ii) Antibiotic susceptibility.** Since SurA, Skp, and DegP decisively shape the composition of the OM, we investigated the impact of their single, double, and triple deletion on antibiotic susceptibility. To this aim, we performed broth microdilution assays with a set of antibiotics relevant for the treatment of infections caused by A. baumannii according to the European Committee on Antimicrobial Susceptibility Testing (EUCAST) (http://www.eucast.org) or to the Clinical and Laboratory Standards Institute (CLSI) (http://em100.edaptivedocs.net/dashboard.aspx) for piperacillin-tazobactam. Additionally, we tested some antibiotics that are not typically used in the treatment of A. baumannii infections (thus no breakpoints were available) as well as vancomycin as an indicator of OM permeability (Table 1). Taken together, we found rather subtle changes in antibiotic susceptibility of the single knockout mutants compared to the wild-type strain. All double mutant strains showed increased susceptibility to a broader panel of antibiotics compared to wild-type strains and single mutants. Interestingly, the Δ*surA*Δ*skp* mutant strain was more susceptible to all tested antibiotics compared to the wild-type strain. However, the most striking finding was that lack of SurA resensitized AB5075 to treatment with the aminoglycoside tobramycin since the MIC for tobramycin dropped below the EUCAST breakpoint for resistance (http://www.eucast.org) in all the mutants lacking SurA. The Δ*surA*Δ*skp*Δ*degP* strain showed an even lower MIC for tobramycin suggesting a synergistic impact of all three chaperones on tobramycin as well as amikacin resistance.

**TABLE 1:**
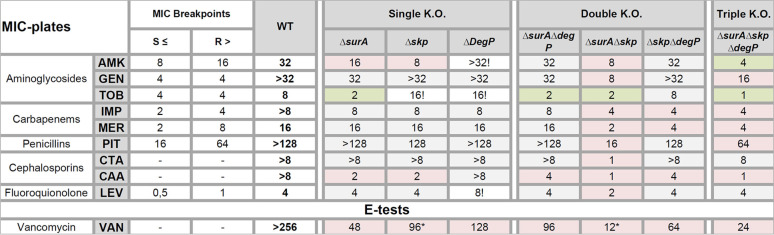
Influence of knockouts on susceptibility against common antibiotics^*a*^

a Antibiotic susceptibility of AB5075 wild type (WT) compared to the periplasmic chaperone deletion mutants was assessed by broth microdilution and Etest. A red background indicates a decreased MIC value that is still above the breakpoint. A green background labels MIC values that dropped below the breakpoint. A gray background indicates a reduction of the MIC of less than 2-fold or unchanged MIC. Increased MIC values are labeled with an exclamation mark. Asterisks mark where colonies appearing in the inhibition zone in Etests. AMK, amikacin; GEN, gentamicin; TOB, tobramycin; IMP, imipenem, MER, meropenem; PIT, piperacillin-tazobactam; CTA, ceftolozan/tazobactam; CAA, ceftazidim/avibactam; LEV, levofloxacin. Gray background, MIC reduced less than 2-fold or unchanged; red background, MIC reduced at least 2-fold; green background, MIC dropped below the breakpoint (if available) according to EUCAST or CLSI in case of PIT; !, MIC increased; *, colonies in zone of inhibition.

With respect to vancomycin, all mutant strains were more sensitive than the wild type. The most pronounced effect was observed in the Δ*surA*Δ*skp* mutant strain. Of note, only with this mutant did we frequently observe some colonies within the zone of inhibition (values labeled with asterisks in Table 1). After regrowth of some of these colonies, we repeated the vancomycin Etest. The isolated clones were sensitive to vancomycin but still more resistant compared to the Δ*surA*Δ*skp* parent strain (32 mg/liter).

All in all, the lack of individual periplasmic chaperones had only a mild effect on antibiotic susceptibility. However, the deletion of *surA*, *skp*, and *degP* synergistically enabled a break of resistance against two of the three tested aminoglycosides, indicating an indirect or even direct role of these chaperones in aminoglycoside resistance and specifically an important role of SurA in tobramycin resistance.

**FIG 4:**
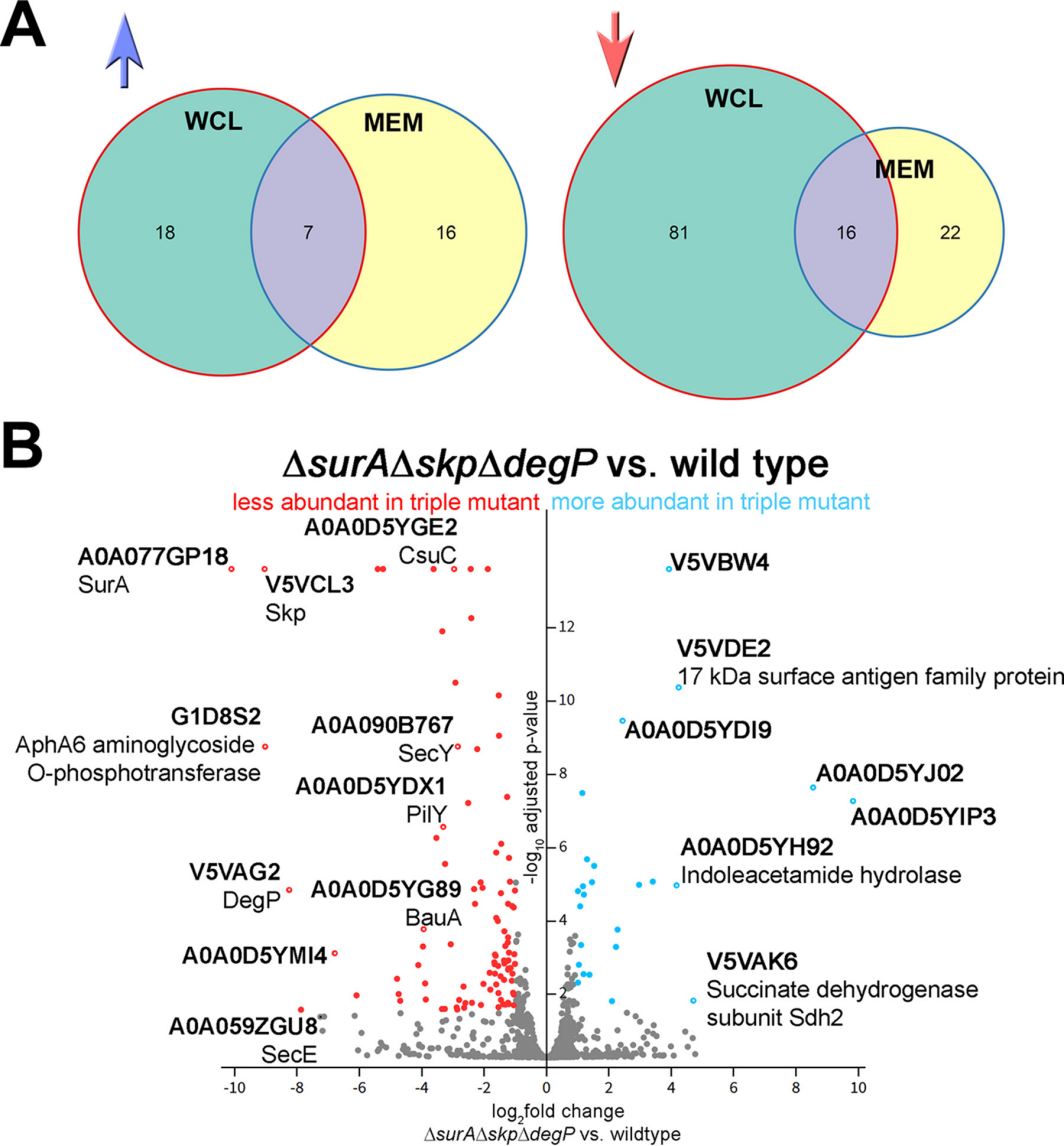
Overview of proteins that showed statistically significant changes in lable-free quantification (LFQ) intensities. (A) Venn diagrams visualize the number of proteins that were less (red arrow) or more abundant (blue arrow) in the samples of the Δ*surA*Δ*skp*Δ*degP* mutant strain compared to wild type. Both, proteins from whole-cell lysates (WCL) or membrane fractions (MEM) fraction are shown. Overlapping areas indicate that proteins were detected in both fractions. (B) Volcano plot showing statistical significance (−log_10_ of *P* values) versus magnitude of change (log_2_-fold change) of all detected proteins in WCL samples. Colored dots indicate statistically significant differences. Gray dots indicate proteins that were not significantly altered in the triple mutant strain compared to wild type. Selected proteins that were mentioned in the article are labeled with a white dot in the center and UniProt ID and protein name (if available).

**(iii) Comparative mass spectrometric analyses of AB5075 wild-type and Δ*surA*Δ*skp*Δ*degP* proteomes.** To identify changes in the proteome of the triple mutant that might help interpret the observed altered phenotypes, we carried out mass spectrometric (MS) analyses of whole-cell lysates (WCL) of the wild-type strain and the triple-knockout mutant lacking SurA, Skp, and DegP. Additionally, we analyzed by MS a membrane-enriched fraction (MEM) to also catch membrane proteins with low abundance. We then performed a differential expression analysis comparing the label-free quantification (LFQ) intensity values of proteins identified in the Δ*surA*Δ*skp*Δ*degP* mutant strain samples to those of the wild-type strain by employing the DEP package (32) (Fig. 4). Results were then filtered for observations that had at least a 2-fold change in mean LFQ intensity and an adjusted *P* value of at least 0.05 (corresponds to [−log10] of a *P* value of 1.30). We found that ~74% of the proteins that showed a statistically significant change of LFQ intensities (160 in total) were less abundant in the triple mutant (119/160) in either of the data sets (WCL or MEM) (Fig. 4; interactive volcano plots for WCL [https://bit.ly/379SM6R] and MEM [https://bit.ly/35kHHNV]). A selection of proteins previously observed to be affected in other species, or being significantly changed in the Δ*surA*Δ*skp*Δ*degP* mutant strain is shown in Table 2. For a comprehensive overview of all proteins, the abundance of which was significantly changed at least 2-fold in WCL and/or MEM, refer to Supplemental file S1.

**TABLE 2:**
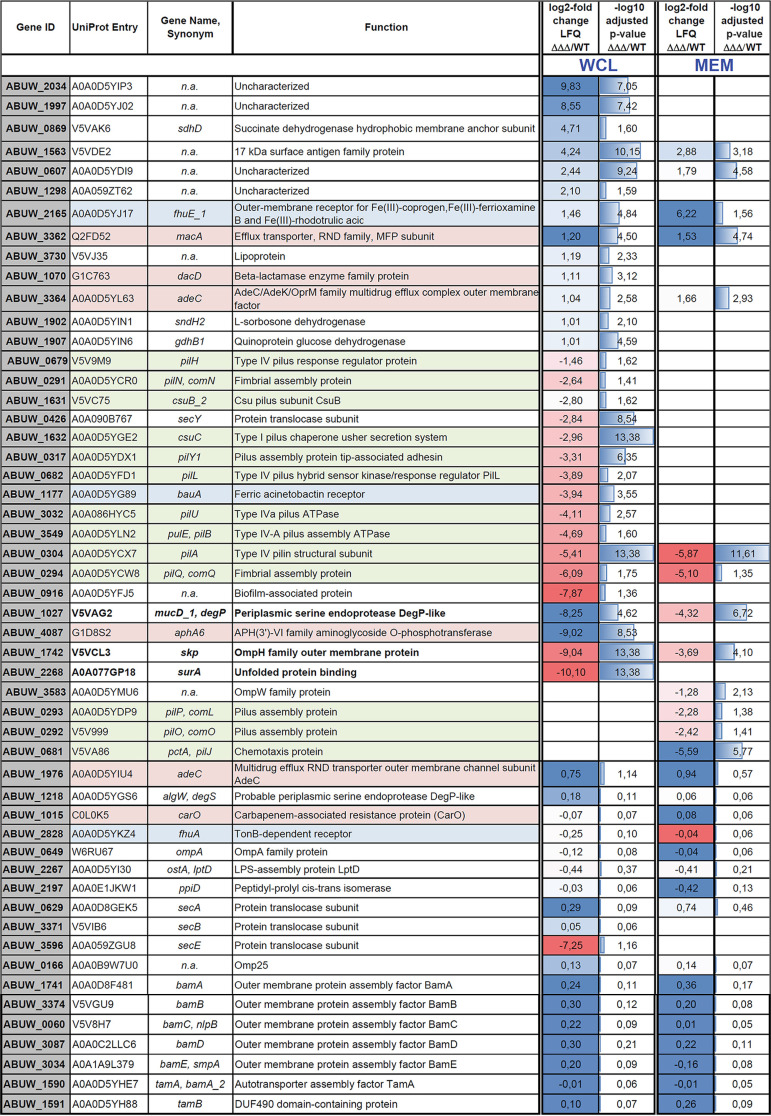
List of selected proteins significantly altered in the Δ*surA*Δ*skp*Δ*degP* (ΔΔΔ) mutant strain compared to the A. baumannii wild-type strain or not showing significant alteration^*a*^

a The latter proteins were included because they are known to be affected by the lack of SurA in other species or might be of interest in other respect. All samples for MS analyses were prepared independently and analyzed in three replicates. The top of the table includes proteins that revealed a statistically significant change (*P* value ≤ 0.05) when comparing the means of LFQ intensities of three sample replicates of WCL or MEM (for details of analysis please refer to Materials and Methods); the bottom includes proteins not showing significant alteration. Log2-fold changes in LFQ intensities of Δ*surA*Δ*skp*Δ*degP*/wild type were calculated for easier comparison. These entries were colored by using the conditional formatting option of Excel, assigning colors according to cell values by using a heat map ranging from blue (high values) over white to red (low values). Additionally, the corresponding −log_10_ adjusted *P* values for all changes are shown. Entries for SurA, Skp, and DegP are boldface. Color code for entries is as follows: green, pil proteins; red, proteins associated with a function in antibiotic drug resistance; blue, proteins involved in iron acquisition; n.a., gene name not available.

Next, we asked whether representatives of the OMP family were affected in the triple mutant strain. Neither of the typically highly abundant OMPs OmpA (W6RU67), Omp25 (A0A0B9W7U0), CarO (C0L0K5), or AdeC (A0A0D5YIU4) showed a significant change. Interestingly, also LptD (A0A0D5YI30) and FhuA (A0A0D5YKZ4), the OM insertion of which is strongly SurA-dependent in E. coli (16), Y. enterocolitica (8), and P. aeruginosa (9), did not exhibit significant changes in protein levels. Our findings thus indicate that the triple knockout does not induce a global reduction in OMP abundance and does not impact specific proteins found to be SurA-dependent in other Gram-negative bacteria. However, we observed a specific and significant reduction of other OMPs, such as the acinetobactin receptor BauA (A0A0D5YG89). Also, several proteins assigned to the subcategory type IV pili (A0A0D5YCR0, A0A0D5YDX1, A0A0D5YFD1, V5V9M9, A0A086HYC5, A0A0D5YLN2, A0A0D5YCX7, A0A0D5YCW8, A0A0D5YDP9, V5V999, and V5VA86), as well as a number of uncharacterized proteins were significantly less abundant. Two components of the type I chaperone-usher pilus system, CsuB (V5VC75) and CsuC (A0A0D5YGE2), were also less abundant in the triple mutant. We also found that the aminoglycoside-inactivating enzyme APH(3′)-VI family aminoglycoside *O*-phosphotransferase (G1D8S2) (33) was below the detection limit in the triple mutant strain, but well expressed in the wild-type strain. This could possibly explain the observed reduced resistance toward tobramycin in the triple mutant.

Another remarkable observation was that the protein translocase subunit SecY (A0A090B767) appeared to be significantly less abundant in the triple mutant. As a part of the general secretion pathway (Sec), SecY is involved in the process of translocation of nascent proteins, including OMPs, into the periplasm (34).

Of course, we cannot discern whether the observed changes are directly or indirectly linked to the lack of SurA, Skp, and DegP.

Next, we searched our data set for proteins that were not or only weakly expressed in the wild type and significantly more abundant in the triple mutant. We identified 25 such proteins in the WCL of the triple mutant strain. Assuming that factors that compensate for the lack of periplasmic chaperones should also act in the periplasm, we then filtered out cytoplasmic proteins. Of the remaining noncytoplasmic candidate proteins many are uncharacterized (A0A0D5YIP3, A0A0D5YJ02, A0A059ZT62, A0A0D5YDI9, and V5VJ35), three are associated with antibiotic resistance (G1C763, Q2FD52, and A0A0D5YL63), three are classified as dehydrogenases of different substrates (A0A0D5YIN6, A0A0D5YIN1, and V5VAK6), one is the outer membrane iron receptor FhuE_1 (A0A0D5YJ17), and one is classified as member of the 17-kDa surface antigen family (V5VDE2). As none of these candidates obviously qualified as periplasmic chaperones we, had a closer look also at the poorly annotated proteins. A0A0D5YIP3 and A0A0D5YJ02 are encoded in the genome of AB5075 several times (3× and 7×, respectively). Their genomic context and BLAST searches suggest they are both phage derived. Performing a copy number variation (CNV) analysis, we found that the genes of both proteins occur in multiple A. baumannii strains and have different copy numbers (Table S5). The CNV varies from 2 to 5 copies for A0A0D5YIP3 and 2 to 9 copies for A0A0D5YJ02. Actually, strain AB5075-UW and very closely related strains have the highest copy numbers, but especially the genes encoding A0A0D5YJ02 are found in more than one copy also in other strains. Furthermore, both genes are present at the same time in the genomes only in a minority of A. baumannii strains.

A0A059ZT62 has been annotated as a l-2,4-diaminobutyrate decarboxylase in the A. baumannii strain NCGM 237. This enzyme is part of the biosynthetic pathway for the production of the polyamine 1,3-diaminopropane, which has been linked to surface-associated motility and virulence of A. baumannii (35). A0A0D5YDI9 and V5VJ35 are lipoproteins of unknown function. V5VDE2 has been annotated in other A. baumannii strains as a glycine-zipper containing OmpA-like membrane domain protein, but this protein is also uncharacterized.

Additionally, we checked for single nucleotide polymorphisms (SNPs) or genomic rearrangements in our mutants (Table S6). The reference strain (AB5075-UW) was highly similar to the wild-type (WT) lab strain (1 synonymous SNP), which was used to generate the mutants. The distribution of the SNPs across the reference genome was homogenous. In the triple mutant (ΔΔΔ), we found 3 SNPs encoding missense variants. Two of them are consistent also with our proteomics data and occur in the hemB gene (ABUW_3022) encoding the delta-aminolevulinic acid dehydratase. The third missense variant affects the methionine adenosyltransferase encoding gene metK (ABUW_2296). Although we performed intense literature searches, we could neither find any connection of the affected genes to the phenotypes we had observed in our study, nor could we find any hints that the variants might compensate for the lack of *surA*, *skp*, and *degP*. In the double mutants, we found 9 SNPs in total, of which 2 are synonymous, 6 lead to missense variants, and 1 leads to a stop within ABUW_0433 encoding a NAD(P)/FAD-dependent oxidoreductase (in the Δ*surA*Δ*skp* mutant strain only). Again, we could neither find any association of these genes with the phenotypes we have observed nor do we have any hints that these variants might facilitate the survival of the double mutants.

Taken together, we found numerous changes in the proteome of the triple mutant strain. However, they do not obviously explain how A. baumannii manages to cope with the absence of SurA, Skp, and DegP. Proteins that were more abundant in the triple mutant strain are either of unknown function, or their suggested function is not associated with specific phenotypes. Therefore, especially the so-far uncharacterized candidates need further investigation, which has been initiated already and will be addressed in the near future. Even if we finally could not answer the question of how A. baumannii can live in the absence of SurA, Skp, and DegP, we investigated how the observed proteomic changes influence specific virulence-associated phenotypes of A. baumannii and translate into overall virulence in an infection model.

**FIG 5:**
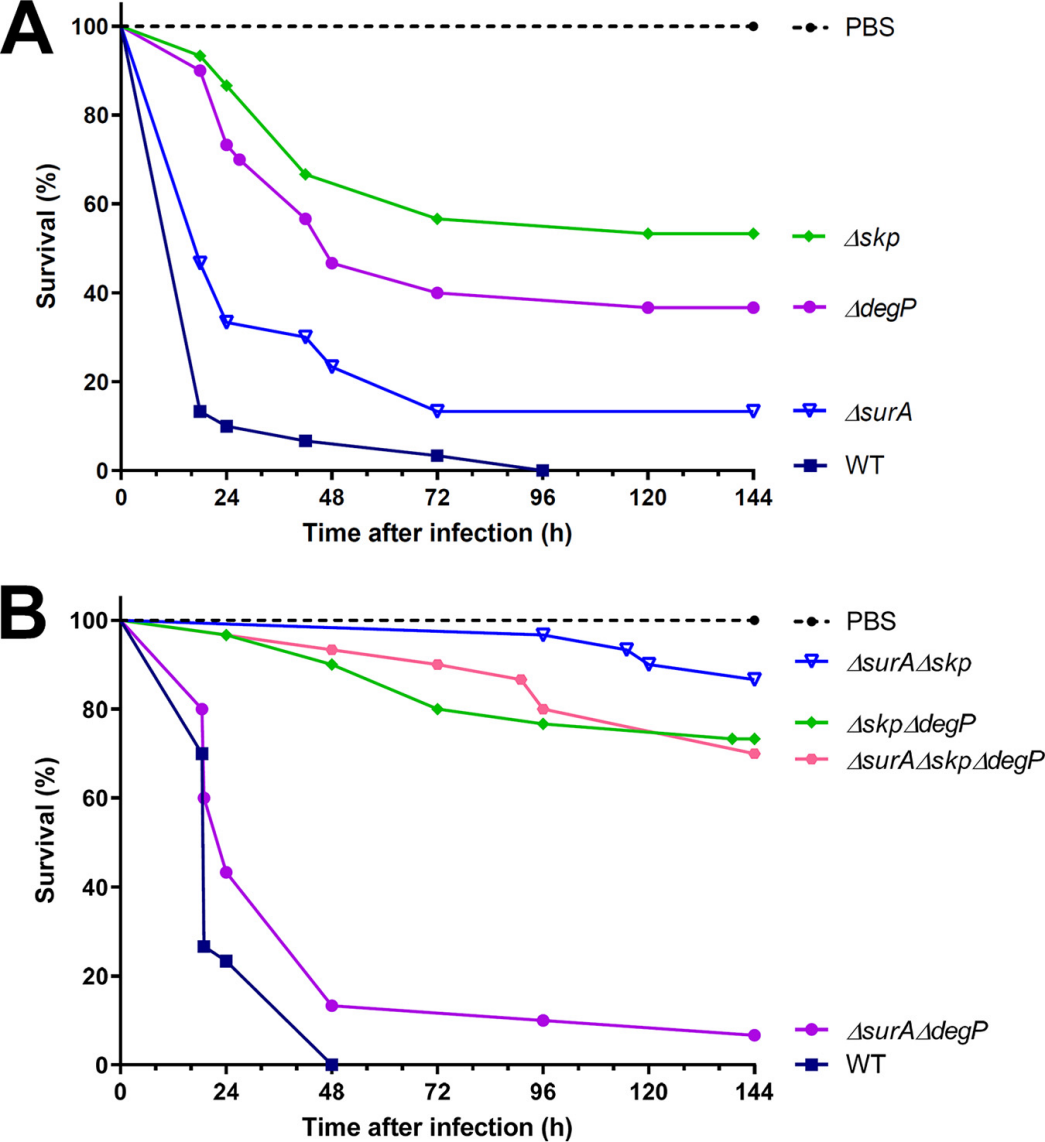
Assessment of virulence of AB5075 wild-type and mutant strains in the Galleria mellonella infection model. The infection of Galleria mellonella larvae per strain was performed to study the role of periplasmic chaperones for virulence. Ten larvae were injected with the indicated strains and incubated at 37°C for 6 days in three independent experiments. Larvae were recorded as dead when no movement occurred after cautious touching with forceps. Survival plots show the combined data for 30 larvae per strain. (A) Survival of single knockout strains compared to wild type (WT). (B) Survival of double and triple knockout strains compared to wild type. Statistical analysis was performed using a log rank test (Mantel-Cox test). A significant difference between wild type and all mutant strains was observed. The *P* value was 0.0233 for Δ*surA*Δ*degP* and for wild type versus Δ*surA* 0.0056 and <0.0001 for wild type versus Δ*skp*, Δ*degP*, Δ*surA*Δ*skp*, Δ*skp*Δ*degP*, and Δ*surA*Δ*skp*Δ*degP*.

**(iv) Virulence.** We assessed virulence of the different strains in the Galleria mellonella infection model. To this end, we injected 30 larvae with 1.1 × 10^5^ to 2.3 × 10^5^ cells of the wild-type strain and the single mutant strains, respectively. The survival of the larvae was monitored over 6 days (Fig. 5A). All larvae infected with the wild type died within 96 h. However, larvae infected with the single mutants had a significantly enhanced survival rate. The deletion of Skp resulted in the highest survival rate (53.3%, *P* < 0.0001) followed by the Δ*degP* (36.7%, *P* < 0.0001) and the Δ*surA* strain (13.33%, *P* < 0.0078), indicating a more critical role of Skp than SurA in virulence. Next, we analyzed the effect of the double and triple knockouts compared to the wild-type strain (Fig. 5B). Please note that the experiments shown in Fig. 5A and B were carried out independently because of technical reasons. Again, 30 larvae were injected with 9 × 10^4^ to 1.12 × 10^5^ cells of the respective strains, and survival at 37°C was monitored for 6 days after injection. All larvae infected with the wild type died within 48 h. The simultaneous knockout of Skp and SurA induced the greatest reduction of virulence (86.7%, *P* < 0.0001). The Δ*skp*Δ*degP* (73.3%, *P* < 0.0001) and the triple-knockout mutant strain (70%, *P* < 0.0001) resulted in comparably enhanced survival rates. However, the growth defect of the Δ*surA*Δ*skp*Δ*degP* mutant strain (see Fig. S4) could also have contributed to its decrease in virulence. The injection of larvae with the Δ*surA*Δ*degP* strain produced the lowest survival rate (6.7%, *P* < 0.0233). Taken together, all strains that lacked Skp either individually or in combination with other chaperones were reduced in virulence, while in contrast to Y. enterocolitica and P. aeruginosa Skp plays only a minor role in virulence.

## DISCUSSION

Given the differential importance of the periplasmic chaperones SurA, Skp, and DegP in E. coli, Y. enterocolitica, P. aeruginosa, and N. meningitidis, and their potential as targets of drugs that reduce the virulence of pathogens and/or resensitize them to antibiotic treatment, we sought to investigate their relevance in MDR A. baumannii. Unexpectedly, single gene knockouts of these periplasmic chaperones in the MDR strain AB5075 (25) caused only mild defects. Analysis of the double knockout strains revealed more pronounced mutant phenotypes, especially in the strains that lacked Skp (see Table 3 for a summary). Nevertheless, all strains were viable unlike what has been published for E. coli questioning the existence of two redundant periplasmic chaperone pathways for OMP biogenesis in A. baumannii as proposed in E. coli (10, 11). Interestingly, like A. baumannii, N. meningitidis also tolerates all combinations of double knockouts. The secondary role of SurA in N. meningitidis could be dictated by specific characteristics in the N. meningitidis SurA protein sequence (18). Compared to its counterpart in E. coli, N. meningitidis SurA actually lacks the first of the two peptidyl-cis/transisomerase (PPI) domains, which has been implicated in binding of peptides resembling unique motifs of OMPs (36). Thus, its absence could possibly change substrate specificity. However, A. baumannii SurA does possess 2 PPI domains and is more similar to E. coli SurA than to the N. meningitidis chaperone (Fig. S5 and Tables S3 and S4). Hence, the different substrate specificity of A. baumannii SurA cannot entirely explain the viability of the double knockouts in A. baumannii as it may in N. meningitidis.

**TABLE 3:**
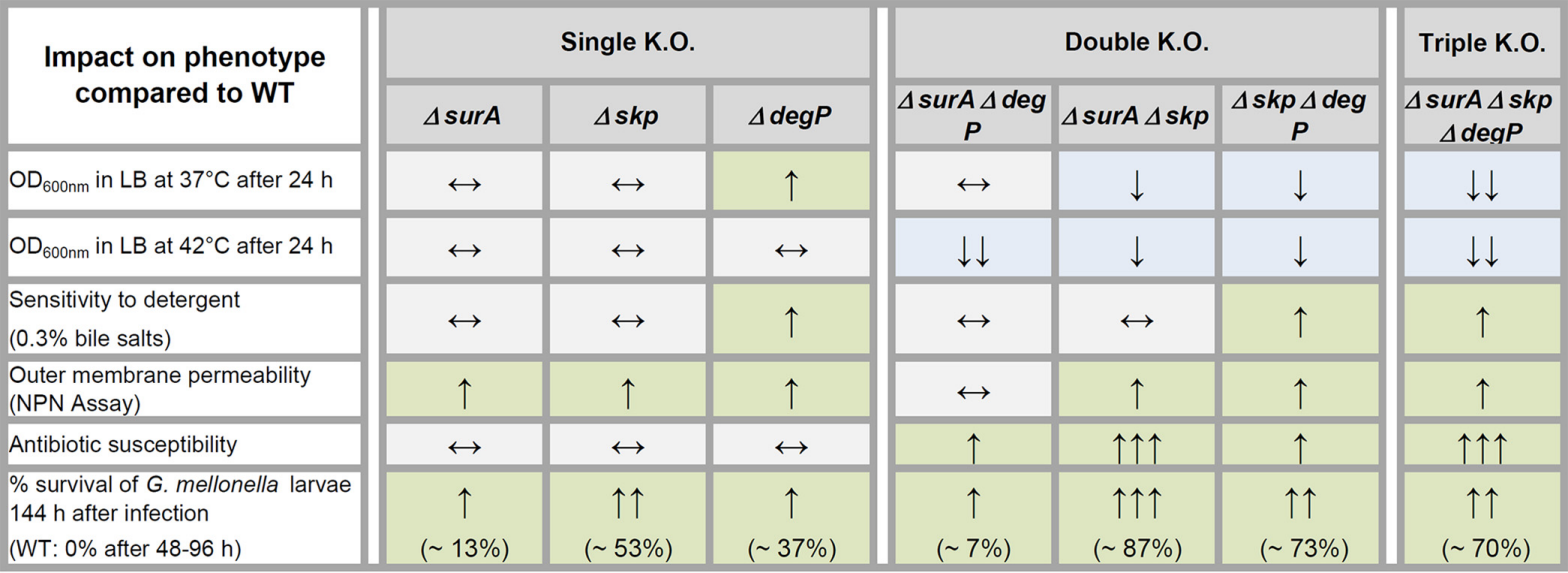
Overview of the altered phenotypes observed in the single, double, and the triple knockout strains compared to the WT strain AB5075^*a*^

a ↑ increased, ↓ reduced, ↔ no change compared to WT.

Surprisingly, even an A. baumannii strain lacking simultaneously SurA, Skp, and DegP was still viable. To our knowledge such a triple periplasmic chaperone knockout has not been created in other Gram-negative pathogens before. Using MS proteome analyses, we did see changes in the abundance of some specific OMPs in the triple mutant but not a general reduction in the majority of β-barrel OMPs (see Fig. 4). In particular, some OMPs previously reported to be strictly SurA dependent such as LptD and FhuA were only slightly affected (Table 2) (8, 9, 16, 17). In E. coli, Y. enterocolitica, and P. aeruginosa deletion of SurA specifically impairs the OM insertion of LptD (8–10, 16). The slightly decreased levels of LptD we observed in the triple mutant indicate that in A. baumannii LptD has to be inserted SurA independently. In E. coli, Y. enterocolitica, and P. aeruginosa, LptD is an essential OMP that in complex with LptE facilitates the assembly of the lipopolysaccharide (LPS) at the surface of the OM (37). Most of the phenotypes, and especially the membrane defects that have been observed in SurA knockout strains of E. coli, Y. enterocolitica, and P. aeruginosa, can presumably be attributed to the defective LPS assembly. Strikingly, N. meningitidis and A. baumannii can live without LPS in their OM (38, 39), which could also explain the minor impact a SurA knockout has on these species.

The proteomic analysis further revealed that the acinetobactin receptor BauA (A0A0D5YG89) was reduced below the detection limit in the triple mutant and also many components facilitated the assembly of type IV pili. As both type IV and type I pili are downregulated under iron limitation (40), this could be a secondary effect caused by impaired uptake of iron due to decreased abundance of BauA in the triple mutant. Further evidence in support of the idea that the triple mutant strain might have a problem with iron acquisition comes from the observation that the OM receptor for Fe(III)-coprogen, Fe(III)-ferrioxamine B and Fe(III)-rhodotorulic acid, FhuE (A0A0D5YJ17), which is induced under iron starvation (41), was significantly more abundant in the mutant strain. We also tested whether the triple mutant displayed any growth defect when cultivated under iron-limiting conditions and found that indeed it has a subtle growth phenotype compared to the wild-type strain (Fig. S6).

Also, the levels of SecY and SecE were significantly reduced in the triple mutants based on the proteomic data. SecG, A, and B instead were not affected. SecY and E are both components of the SecYEG preprotein translocase in E. coli, facilitating protein translocation across the inner membrane (34). The effect of a reduction of SecY levels has not been analyzed before for A. baumannii, but it is known that *secY* is an essential gene also in A. baumannii (26, 42, 43). SecA is known to function as an ATPase driving protein precursor through the SecYEG protein translocation channel (44). Recently, Fiester et al. (45) generated a viable *secA* mutation in A. baumannii 19606T by insertion of a transposon at its 3”end. In contrast to the deletion of the E. coli counterpart, the *secA* mutation was not lethal but resulted in impaired protein translocation, iron acquisition, and virulence. Few reports even suggest the existence of SecA-only channels that act independently from SecYEG (46). Considering these findings, the significantly decreased expression of SecY could be contributing to the reduced abundance of several Sec-dependent proteins involved in virulence and antibiotic resistance in the triple-knockout strain. We can only speculate whether this represents a strategy to reduce periplasmic stress by restricting entry of OMPs or a secondary effect.

A common theme of our analyses was that different combinations of knockouts mostly had differential impact depending on the phenotype studied. Nevertheless, all double and the triple knockout mutants displayed a growth phenotype at 42°C and increased antibiotic susceptibility (especially for aminoglycosides). Skp seems to be the main driver of virulence in the Galleria mellonella infection model. Also, the assays probing membrane integrity (NPN, bile salts) indicated Skp as being the most important factor for maintaining OM integrity. In contrast, we found that the efflux of NPN was significantly impaired only in a strain lacking DegP. DegP has previously been linked to resistance to the cationic antimicrobial peptide protamine (47) as well as to high-salt concentrations (31), presumably by indirectly contributing to the assembly of tripartite efflux pump systems. This could also explain our finding with regard to NPN efflux, especially as NPN has already been described as a substrate of RND-type efflux pumps (48).

The observed increase in antibiotic susceptibility in the triple mutant may, at least in part, be linked to the absence of the APH(3′)-VI family aminoglycoside *O*-phosphotransferase (G1D8S2), an enzyme inactivating aminoglycosides (33). The encoding gene *aph6* (encoded on the ~84 kb plasmid p1AB5075 [26]) is a transposable element (49) and was lost in the triple deletion mutant as revealed by genome sequencing analysis. This explains the absence of the Aph(3′)-VI protein in our MS data set. However, the *aph6* gene was still present on p1AB5075 of all double knockout strains, so their increased sensitivity toward treatment with aminoglycoside antibiotics presumably has a different confounder. Additional factors and mechanisms, however, contribute to the resistance to treatment with tobramycin (26, 50, 51). Among the core genome genes associated with high-level tobramycin resistance in AB5075 were several genes associated with envelope biogenesis (50), particularly the two PPIases *ppiB* and *ppiD* as well as *surA* and *ppiA*, although less consistently. These findings are consistent with our data and suggest a specific role of SurA and a more general role of PPIases in tobramycin resistance.

All in all, our findings raise the question of how OMPs can be inserted into the OM of A. baumannii in the absence of the three best-studied periplasmic chaperones (3). A search for paralogues of SurA, Skp, and DegP essentially yielded two proteins, namely, PpiD (A0A0E1JKW1) and DegS (A0A0D5YGS6) (Table S4). The similarity of PpiD to SurA arises from the high sequence identity of a PPI domain present in PpiD. Moreover, homologous N-terminal and C-terminal domains of SurA are also present in PpiD. Nevertheless, they are conserved to a lower extent. In E. coli overexpression of PpiD can rescue the synthetic lethality of the *surA skp* null mutant (52). This rescue is dependent on those domains of E. coli PpiD that are homologous to the SurA core domain (consisting of the N- and C-terminal domains and comprising the chaperone function) (36) but independent of the PPIase domain. It was also shown that E. coli SurA and PpiD possess partially overlapping substrate specificity (53). Therefore, we cannot rule out that PpiD might compensate for the chaperone function of SurA. The potential paralogue for DegP, DegS, senses misfolded polypeptides and in contrast to DegP, is exclusively responsible for the proteolytic inactivation of the anti-sigma factor RseA (54). At least in E. coli, DegS cannot compensate for the heat-sensitive phenotype of a protease-deficient DegP or the knockout of *degP*. As the A. baumannii Δ*degP* strain displays mutant phenotypes clearly associated with DegP deficiency, we do not assume that DegS compensates for the lack of DegP in A. baumannii. Multiple attempts to generate s*urA*, s*kp*, *degP*, *degS*, as well as *surA*, *skp*, *degP*, and *ppiD* quadruple deletion mutants failed. In contrast, we were able to delete a different gene, e.g., ABUW_1852, ABUW_1948, or ABUW_0607, in the *surA*, *skp*, and *degP* knockout strain, indicating that the triple mutant can be genetically manipulated. As the further deletion of DegS and PpiD is possibly lethal, or at least undermines cell physiology to an extent that cells cannot survive the selection process, we assume they do play a role in OMP biogenesis in A. baumannii. For DegS, we assume that this is most likely an indirect effect, resulting from the disturbance of the initiation of an appropriate sigma-dependent envelope stress response which is essential to the survival of the triple mutant strain. However, further studies will have to elucidate the role of PpiD in OMP biogenesis in A. baumannii in more detail.

**FIG 6:**
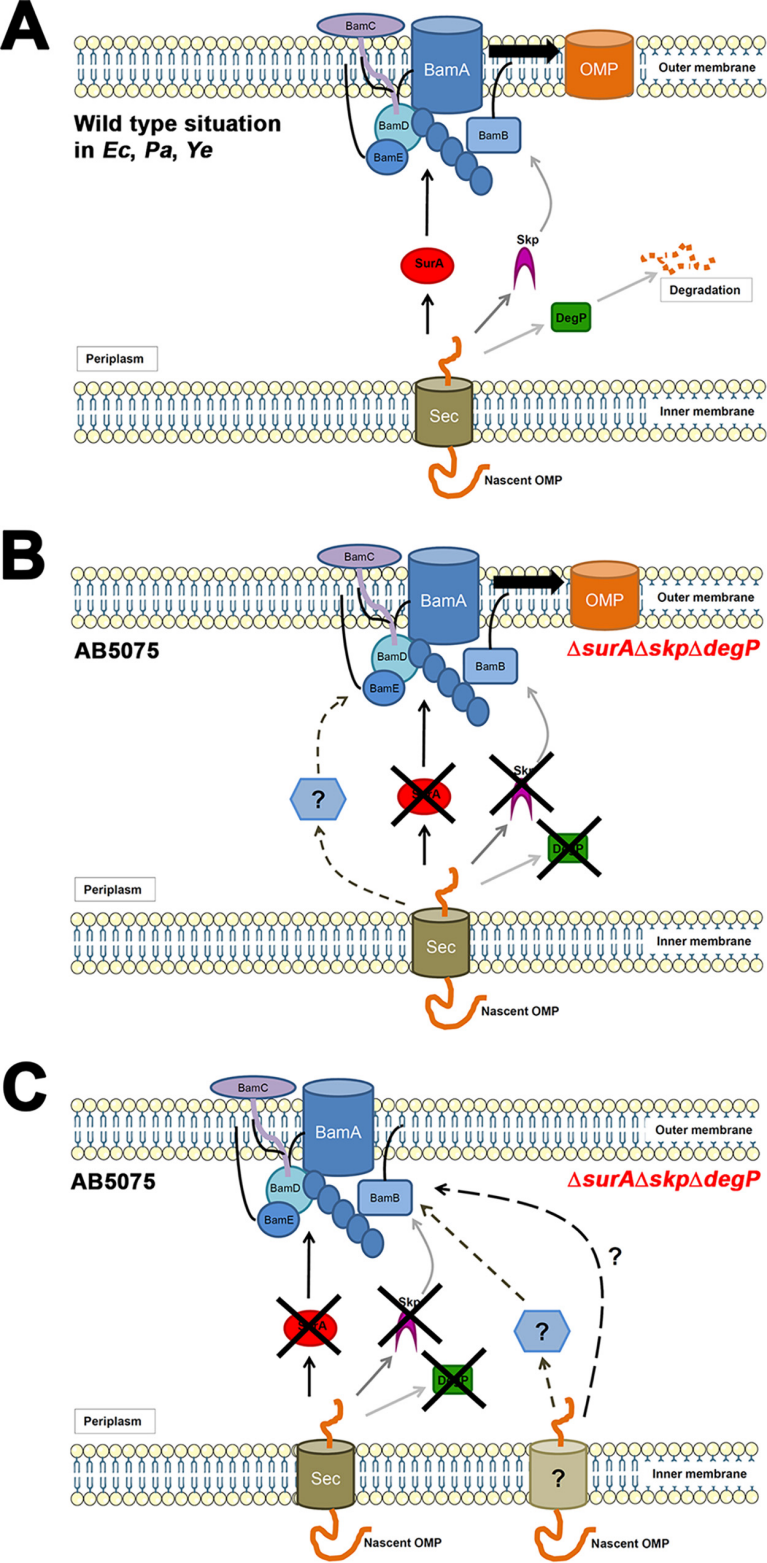
Canonical periplasmic chaperone network in Gram-negative bacteria and possible compensation strategies of AB5075 in the absence of SurA, Skp, and DepP. (A) OMP biogenesis in the wild type E. coli, P. aeruginosa, and Y. enterocolitica. After translocation into the periplasm though the secretory export machinery (Sec), nascent OMPs are guided through the periplasm by SurA or Skp/DegP. Misfolded or aggregated OMPs are degraded by the protease activity of DegP. After passage through the periplasm, the BAM complex facilitates insertion of the OMPs into the OM. (B) AB5075 possesses additional periplasmic factors that can transport OMPs to the BAM complex in the absence of SurA, Skp, and DegP. (C) AB5075 employs a pathway independent of SecY, SurA, Skp, and DegP to insert OMPs into the OM. Certain elements such as membrane bilayers were drawn using templates from Servier Medical Art (https://smart.servier.com/).

As of today, we can only put forward alternative hypotheses for the unique ability of A. baumannii to survive in the absence of the three main periplasmic chaperones, which will have to be tested in the near future (Fig. 6). One explanation for our findings, deviating from what is known for E. coli, P. aeruginosa and Y. enterocolitica (Fig. 6A), would be the existence of periplasmic factors that are at least partially redundant to SurA, Skp, and DegP (Fig. 6B). As such, their function would be to escort nascent OMPs across the periplasm and deliver them to the Bam complex for insertion. These factors presumably cannot handle distinct substrates as efficiently as SurA, Skp, or DegP do. This could explain why the triple knockout is viable but does have defects. In addition, the existence of these chaperones could explain why the abundance of several OMPs, previously shown to rely on SurA for insertion, remains unchanged. Also, transcriptional upregulation of these OMPs as part of a stress response may play a role in the maintenance of protein levels, but still, there has to exist a pathway that facilitates their insertion independently of SurA, Skp, and DegP. Some yet uncharacterized proteins as well as PpiD may qualify for this role. To overcome the reduced SecY protein levels, a pathway acting independently of SecY and SurA, Skp, and DegP (Fig. 6C) might exist. Some authors have suggested the existence of supercomplexes bridging the periplasm through direct interaction of Sec components and the BAM complex (55–57). Their models do not comply with our findings as they involve SecY and SurA. However, if organized differently and able to prevent OMP aggregation and facilitate OM insertion, a supercomplex could provide a mechanism to reduce the importance of periplasmic chaperones. While the mechanisms A. baumannii employ to get along without the main periplasmic chaperones SurA, Skp, and DegP remain elusive, the novelty of our study is in demonstrating that A. baumannii can actually get along without them and that findings in one pathogenic species cannot automatically be transferred to another. This has important implications for future strategies aimed at treating infections by multidrug-resistant A. baumannii.

## MATERIALS AND METHODS

### Cultivation of bacteria in liquid and on solid media.

AB5075 was routinely grown in LB (Miller) medium without supplements. Overnight cultures were inoculated from glycerol stocks and grown overnight without shaking at room temperature (RT) to keep the strain in an opaque state (58). Every culture was routinely tested for the presence of a capsule as an indicator of the strain being in an opaque state by a Maneval’s stain before being used for experiments. For growth on LB agar (e.g., after mutagenesis), bacteria were incubated at 37°C for 18 h with supplements if indicated. Strains and supplements used are listed in Table S1.

### Generation of knockout mutants of AB5075.

To generate markerless single gene deletion mutants, a plasmid harboring a knockout cassette having an appropriate selection marker (Tellurite^Res^) was created. For amplification of the up- and downstream regions of the gene to be deleted, genomic DNA of AB5075 was isolated using the QIAamp DNA minikit (Qiagen). The flanking regions of genes of interests comprising ~1,000 bp of the 3′- and 5′-ends and a scar sequence encoding a small peptide of 30 aa (first 15 and last 15 amino acids, including the start and stop codon of the gene to be deleted) were amplified by PCR using genomic DNA as the template. The plasmid pVT77 (27) synthesized by GenScript (GenBank: KX397287.1) was linearized also by PCR and purified using the Promega Wizard SV kit (Promega). Amplified up- and downstream fragments were fused with the linearized and purified plasmid pVT77 by Gibson assembly. Successful insertion of the knockout (KO) cassette was verified by PCR amplification and sequencing of the insert region. All oligonucleotides used for the described PCR amplifications are listed in Table S2.

Next, AB5075 was transformed by exploiting its capacity to take up DNA while moving along a surface. Therefore, a three-streak plate of AB5075 was prepared and incubated overnight at 37°C. For transformation, the knockout plasmid to be transformed (total amount, 4 to 8 μg) was adjusted to a volume of 20 μl in sterile phosphate-buffered saline (PBS) (Gibco) in a 1.5-ml reaction tube. Then, a single colony of AB5075 was picked from the plate prepared the day before using a sterile pipette tip and resuspended in 20 μl knockout plasmid DNA in PBS. The DNA/AB5075 mixture was pipetted up and down several times using a sterile 100-μl pipet tip. Finally, a motility agar plate (5 g/liter tryptone, 2.5 g/liter of NaCl, and 5 g/liter of agarose) was inoculated by stabbing and pipetting 2 μl of the mixture into the agar several times. Plates were closed with a lid, sealed with parafilm, and incubated right side up for 18 h at 37°C. The next day, 1 ml PBS was used to wash off the colonies growing on the surface of the agar. Another 1 ml PBS was used to harvest the cells from the space between plate and agar by stabbing into the agar and dispensing the PBS several times to harvest as much of the bacteria as possible. After harvest, 100 μl bacterial suspension was plated onto LB agar containing tellurite (30 mg/liter for pVT77) (Sigma-Aldrich) as a selective agent. The remaining cells were spun down in a tabletop centrifuge (5,000 × *g* for 5 min) (Eppendorf 5417R, rotor F45-30-11), and the supernatant was discarded. Pelleted bacteria were resuspended in 100 μl PBS and spread onto a second LB agar plate also containing tellurite as a selective agent. Both plates were incubated upside down for 18 h at 30°C. The next day, single colonies were picked to perform colony PCR using the oligonucleotide pairs listed in Table S2 to detect whether the KO plasmid had been taken up. Colonies that tested positive for the presence of the KO plasmid were used to inoculate 2 ml fresh LB broth containing 1 mM IPTG (Peqlab) (without further supplements) to induce expression of the heterologous thymidine kinase encoded on the pVT77 plasmid. Thymidine kinase triggers cytotoxicity in the presence of 3′-azido-3′-deoxythymidine (AZT) (Jena Bioscience). Then, bacteria were allowed to grow for 3 h at 37°C with shaking. Afterwards, bacteria were streaked on selective agar (containing 200 μg/ml AZT) using a 10-μl inoculation loop and incubated overnight at 37°C. To test whether the target gene had been replaced by the KO cassette via homologous recombination, a PCR was carried out using oligonucleotide pairs flanking the corresponding gene using genomic DNA as a template (Table S2). Colonies that tested positive for the deletion of the target gene were additionally tested with another colony PCR using an oligonucleotide pair where the 5′ primer was placed upstream of the target gene and the 3′ primer was placed in the deleted region (Table S2). No PCR product was detected in colonies where the KO was successful. Additionally, the regions containing the gene deletion were verified by sequencing. For the double and triple KO strains, a qualitative RT-PCR was also carried out to test for the presence of mRNA transcripts.

### Isolation of genomic DNA.

Genomic DNA, used for the validation of gene deletions as well as for sequencing, was isolated as follows: 5 ml overnight culture was prepared at 37°C and with shaking at 200 rpm. DNA isolation was performed using the QIAamp DNA minikit (Qiagen) according to the manufacturer’s instructions. Genomic DNA was stored at −20°C until use.

### Library preparation, sequencing, and genome assembly.

Libraries for Illumina sequencing were prepared with Nextera DNA Flex Library Prep kit (Illumina), using as input 500 ng of genomic DNA for each mutant strain. Sequencing was performed on an Illumina MiSeq sequencer using a MiSeq v2 reagent kit (300 cycles) in paired-end mode (2 × 150). In order to verify the expected genome edits in the deletion mutants, we performed *de novo* assemblies using the nf-core/bacass pipeline (59). The gene deletions in each strain were validated by inspecting the respective genomic regions in the assemblies.

### Isolation of RNA and RT-PCR.

For RNA isolation 5 × 10^9^ bacteria were harvested from an overnight culture and resuspended in 1 ml TRIzol Reagent (Thermo Fisher Scientific). RNA isolation and DNase digestion were carried out as described previously (9). The resulting RNA was stored at a concentration of 0.1 μg/μl in RNA storage solution (Invitrogen) at −80°C until use. To carry out RT-PCR, RNA was thawed on ice and diluted 1:10 using RNase-free water (Ambion). mRNA expression of mutant strains in comparison to wild type was analyzed qualitatively by RT-PCR using the QuantiFast SYBR green qRT-PCR kit (Qiagen) according to the manufacturer’s instructions. The resulting PCR products were analyzed by agarose gel electrophoresis and staining of the gel with SYBR Safe (Thermo Fisher Scientific). Primers used are listed in Table S2.

### Generation of pure stocks of opaque cells.

AB5075 can undergo a phase switch that is induced by a high density of cells in the growing culture. This phase switch is associated with a number of phenotypic changes that also influence virulence (58). To generate homogenous opaque state glycerol stocks, a mixed culture was plated on 0.5× LB agar as described in Tipton et al. (58) and grown overnight at 37°C. Essentially, opaque colonies were identified by oblique lighting, and single colonies were used for a three-phase streak on LB agar plates. After 6 to 8 h of growth at 37°C, several single opaque colonies were picked using a dissecting microscope and used to inoculate 2 ml LB medium each. The cultures were then grown overnight at RT. Purity was again assessed by streaking on 0.5× LB agar plates. The remaining cultures were used to prepare glycerol stocks that were flash-frozen and stored until use at −80°C. Only cultures that had an estimated purity of 99% or greater were used for further experiments. Purity was additionally assessed by performing a Maneval’s capsule stain. For the following experiments, overnight cultures were grown at RT and without shaking to obtain AB5075 wild type and the regarding mutant strains in the opaque phase only. Regularly, the opaque state was confirmed by plating the cells on 0.5× LB agar as well as performing Maneval’s capsule stains.

### Maneval’s capsule stain.

A single colony was resuspended in 10 μl PBS. Five microliters of this suspension were pipetted onto the left side of a glass slide for microscopy. A drop of 1% Congo red solution (Carolina) was added and mixed with the bacteria. Then, a smear was prepared by evenly distributing the mixture across the entire slide using a clean glass slide. The smear was allowed to air dry. Next, 3 drops of Maneval’s stain (Carolina) were placed on parafilm and the slide was placed upside down into the staining solution. After a 2-min incubation at RT, excess staining solution was removed by tilting the slide on Whatman paper. The slide was then air-dried, embedded with Roti-Histol (Carl Roth), and sealed with a coverslip. Samples were then analyzed with an upright light microscope using the 63× lens and oil immersion. Only capsule-positive bacteria were characterized by a distinct halo around the bacterial cells.

### Growth curves.

Growth curves were recorded to monitor potential differences in growth between the wild-type strain and the knockout mutants. First, overnight cultures were prepared. Next day, the OD_600nm_ was measured and cultures were adjusted to 1 × 10^7^ cells/ml using fresh LB. In a 24-well plate (Greiner Bio-One), 1 ml of bacterial suspension was added per well. Plates were incubated with a lid and with shaking at 300 rpm at 37°C or 42°C for 14 or 24 h, as indicated. All growth curves were recorded in triplicates and three independent experiments using an Infinite PRO 200 plate reader (Tecan). To test for growth under iron-limiting conditions, 0.2 mM 2,2′-bipyridyl (BiP) was added to the medium.

### Bile salts assay.

Five ml or milliliters of overnight cultures was grown at RT without shaking. The next day all samples were adjusted to 1 × 10^7^ bacteria/ml using PBS and a 10-fold serial dilution using PBS was prepared in a 96-well plate. Five microliters of each sample and dilution was spotted on LB agar plates containing 0.3% bile salts (Sigma-Aldrich). Plates were incubated right side up overnight at 37°C, and pictures were taken the next day using a digital camera. The bile salts assay was performed three times.

### NPN uptake and efflux assay.

To assess OM permeability and efflux activity of A. baumannii mutant strains, an NPN assay was carried out based on protocols by (29, 30). Overnight cultures of opaque strains were grown without shaking and at room temperature. Cells were harvested by centrifugation and washed twice with KPO_4_-MgCl_2_ buffer (20 mM KPO_4_, 1 mM MgCl_2_) at pH 7. Using the same buffer, cell suspensions were adjusted to 4 × 10^8^ cells per ml. Nine-hundred ninety microliters of the bacteria suspensions was transferred to 2-ml Eppendorf tubes for each strain. Ten microliters of a 10 mM CCCP in DMSO stock solution was added to the samples and incubated for 30 min at room temperature in the dark. CCCP deenergizes the cells and thereby inhibits efflux pump activity. Then, samples were washed twice with 2 ml KPO_4_-MgCl_2_ buffer and finally resuspended in 990 μl. Next, cells were loaded with NPN by adding 10 μl of 1 mM NPN in ethanol and an incubation step for 15 min at room temperature in the dark. Finally, 90 μl of each sample was added to a flat-bottom, black, 96-well plate in triplicates. Fluorescence was measured in a Tecan plate reader for 30 s in 5-s intervals (excitation, 340 nm; emission, 410 nm; ex/em slit width, 5 nm) (shows NPN uptake into cells, indicative of OM barrier integrity). At the 30-s time point, 10 μl of glucose (500 mM in KPO_4_-MgCl_2_ buffer) was injected per well via the injector system of the plate reader and fluorescence intensities were recorded for 60 s at 5-s intervals (shows the reduction of fluorescence upon reenergizing the efflux pumps, i.e., the capacity of strains to expel the NPN dye). Hereby, samples were measured one after the other. Pretests had shown that there is no significant effect on NPN uptake or efflux between identical samples that were measured either at first or at last on one plate. Wild-type cells as well as a strain with disturbed AdeABC type efflux pump expression have been used as controls. Three independent assays were performed. Relative fluorescence was calculated by using the AB5075 wild-type strain as a reference. Significant differences were analyzed by a one-way ANOVA analysis.

### Antibiotic susceptibility testing with broth microdilution.

To obtain an AB5075 culture in opaque state, 5 ml of overnight cultures was inoculated from a frozen opaque stock and grown without shaking at RT. Bacteria were harvested by centrifugation for 5 min at 5,000 × *g* (Heraeus Multifuge 3 S-R, rotor 6445), resuspended in 500 μl 0.9% NaCl and adjusted to a 0.5 McFarland standard. To prepare the inoculum for antibiotic susceptibility testing (AST), 62.5 μl of the suspension was mixed with 15 ml of cation-adjusted Mueller-Hinton Broth (Biotrading). Micronaut-S AST plates (MERLIN Diagnostika) were inoculated with 100 μl of bacteria per well according to the manufacturer’s instructions. Plates were sealed with a nonbreathable seal (MERLIN Diagnostika) and incubated for 18 h at 37°C. Subsequently, the seal was removed and the OD_600nm_ was determined using an Infinite PRO 200 plate reader (Tecan). Additionally, plates were visually inspected for growth using a special mirror system.

### E-tests for vancomycin.

As described above, bacteria were adjusted to a McFarland standard of 0.5. Using a sterile cotton swab, the bacteria suspension was distributed evenly on a Mueller-Hinton Broth agar plate (Oxoid). Then, a vancomycin Etest stripe (Liofilchem) was placed in the center of the agar plate and the plate was incubated right side up for 18 h at 37°C. After that, the inhibition zones were measured. The assay was repeated in two independent experiments.

### Preparation of samples for mass spectrometric analysis.

To examine changes in the protein expression of the Δ*surA*Δ*skp*Δ*degP* mutant strain compared to the wild type, an MS analysis was performed, using whole-cell lysates (WCL). For the generation of WCL, the OD_600nm_ of 20 ml of overnight cultures of each strain was measured in a 1:20 dilution using LB. After centrifugation for 5 min at 5,000 × *g* (Heraeus Multifuge 3 S-R, rotor 6445), the supernatant was removed. Next, 100 μl sterile distilled water was added per OD_600nm_ of 0.1 of the diluted sample, and the protein concentration was determined using the Pierce BCA protein assay kit (Thermo Fisher Scientific). Afterward, 100 μl 4× Lämmli buffer (Bio-Rad) per OD_600nm_ of 0.1 containing 10% β-mercaptoethanol (AppliChem) was added. The samples were boiled at 95°C for 5 min. Thirteen micrograms of protein per lane was loaded on a 10% Mini-PROTEAN TG Precast Protein Gel (Bio-Rad), and the samples were allowed to enter the gel by applying a current of 100V for 2 min. Next, the gel was stained with Roti-Blue Colloidal Coomassie Staining solution (Carl Roth) containing 20% methanol (AppliChem).

### Preparation of membrane fractions.

For a more precise proteomic analysis, an additional MS analysis was performed using membrane fractions. Subcellular fractionation was performed as described before (60) with slight modifications. For each strain, 50 ml overnight culture was prepared at RT without shaking. Cells were pelleted by centrifugation for 5 min at 5,000 × *g* (Heraeus Multifuge 3 S-R, rotor 6445). After one single washing step with sterile PBS, cells were resuspended in 500 μl resuspension buffer (0.2 M Tris, 1 M sucrose, and 1 mM EDTA, pH 8.4). Additionally, resuspension buffer was supplemented with 1 mg/ml lysozyme and protease inhibitors (Sigma-Aldrich). The suspension was incubated for 10 min at RT, whereby after 5 min 3.2 ml distilled water was added. By centrifugation for 45 min at 16,000 × *g* (Beckman Coulter Optima LE-80K, rotor: SW 41 Ti) and 4°C, the membrane fraction was separated from other remaining components of the cell. The harvested pellet was resuspended in 2.5 ml lysis buffer (10 mM Tris, 1 mM MgCl_2_, 5 mM EDTA, and 0.2 mM DTT, pH 7.5) and lysed using a french press (Thermo Fisher Scientific). Unlysed cells were removed by centrifugation for 10 min at 5,000 × *g* (Heraeus Multifuge 3 S-R, rotor: 6445) at 4°C. By another centrifugation step at 290,000 × *g* (Beckman Coulter Optima MAX-XP, rotor: TLA 100.3) for 60 min at 4°C, the membrane fraction was pelletized and separated from the cytosolic fraction. The pellet was resuspended in 2 ml extraction buffer (50 mM Tris, 10 mM MgCl_2_, 2% TritonX-100) and incubated for 30 min at 4°C. Then, the suspension was centrifuged for 40 min at 84.000 × *g* (Beckman Coulter Optima MAX-XP, rotor TLA 100.3) at 4°C, followed by 3 washing steps with water and centrifugation for 20 min at 84.000 × *g* (Beckman Coulter Optima MAX-XP, rotor TLA 100.3). The final pellet containing the membrane fraction was resuspended in 100 μl water and prepared for MS analysis as described before.

### Nano-liquid chromatography-tandem mass spectrometry analysis and data processing.

Nano-liquid chromatography-tandem mass spectrometry (nano-LC-MS/MS) analysis and data processing were essentially carried out as described in Klein et al. (9) with the following modifications: peptide mixtures were separated using either a 57 or 127 min segmented gradient (10-33-50-90%) of HPLC solvent B (80% acetonitrile in 0.1% formic acid) in HPLC solvent A (0.1% formic acid) at a flow rate of 200 nl/min. The 7 or 12 most intense precursor ions were sequentially fragmented in each scan cycle using higher energy collisional dissociation (HCD) fragmentation. In all measurements, sequenced precursor masses were excluded from further selection for 30 s. The target values for MS/MS fragmentation were 10^5^ charges and 3 × 10^6^ charges for the MS scan. MaxQuant software package version 1.6.7.0 was used. Database search was performed against a target-decoy Acinetobacter baumannii database obtained from UniProt, containing 3,839 protein entries and 286 commonly observed contaminants. Endoprotease trypsin was defined as protease with a maximum of two missed cleavages. Oxidation of methionine and N-terminal acetylation were specified as variable modifications, and carbamidomethylation on cysteine was set as fixed modification. Initial maximum allowed mass tolerance was set to 4.5 ppm (ppm) for precursor ions and 20 ppm for fragment ions. The iBAQ (Intensity Based Absolute Quantification) and LFQ (Label-Free Quantification) algorithms were enabled, as was the “match between runs” option (61, 62).

### Differential expression analysis of proteomics data.

The analysis was performed with the DEP package ver 1.10.0 (32) in R, using three replicates for each strain. The default workflow was applied, using filtering for proteins that have a maximum of one missing value in at least one condition, variance-stabilizing transformation with the vsn package ver 3.56.0 (63), and MNAR imputation of missing values using the imputeLCMD package ver 2.0 (64). Differential enrichment analysis was performed with the limma package ver 3.44.3 (65), and the volcano and upset plots were generated with the ggplot2 ver 3.3.2 (66) and UpSetR ver 1.4.0 (67) packages, respectively. Interactive volcano plots, available at https://bit.ly/379SM6R (WCL) and https://bit.ly/35kHHNV (MEM), were produced with the Glimma package ver 1.16.0 (68).

### Galleria mellonella infection.

Galleria mellonella larvae were infected to examine the virulence of the knockout mutants in comparison to the wild-type strain. Five milliliters of overnight culture of each strain was prepared at RT without shaking. Bacteria were harvested by centrifugation for 5 min at 5,000 × *g* (Heraeus Multifuge 3 S-R, rotor: 6445). The supernatant was discarded and bacteria were resuspended in 5 ml PBS. The OD_600nm_ of overnight cultures was measured and cells were adjusted to 1 × 10^7^ bacteria/ml using PBS. Ten microliters of the bacteria suspension was injected into 10 Trularv larvae (BioSystems Technology) per strain using a microfine insulin syringe (BD Bioscience). After infection, the larvae were incubated at 37°C for 6 days and inspected regularly. Larvae were defined to be dead when no movement occurred after touching them gently several times with forceps. To determine the actual inoculum, a 10-fold serial dilution of the suspension used for infection of larvae was prepared using sterile PBS. One hundred microliters of the dilutions 10^4^, 10^3^, and 10^2^ was plated on LB agar and incubated overnight at 37°C. The next day, colonies were counted and the actual infection dose was calculated. In total, in six independent experiments, 30 larvae per strain were infected with 9 × 10^4^ to 2.3 × 10^5^ bacteria. As a negative control, larvae were injected with 10 μl PBS only. For statistical analysis, a log-rank test (Mantel-Cox test) was performed.

### Single nucleotide polymorphism analysis.

The SNP analysis was performed using the software snippy ver 4.6.0 (69) and previously calculated assemblies. Snippy was used with the default settings, and as a reference, the Acinetobacter baumannii strain AB5075-UW was used.

### Copy number variation analysis for A0A0D5YIP3 and A0A0D5YJ02.

For the copy number variation analysis, the genes for the two proteins were extracted from Uniprot (70) and a BLASTn search (71) was performed for each gene independently. The BLASTn search was restricted to Acinetobacter baumannii. The resulting hits were checked for overlaps and summarized for each reference strain for all genes.

### Data availability.

**(i) MS data.** The data set of the LC-MS/MS analysis for determination of WCL and OM composition of the investigated bacterial strains can be found in the ProteomeXchange Consortium via the PRIDE (72) partner repository with the data set identifier PXD022004 (http://proteomecentral.proteomexchange.org/cgi/GetDataset) and data set ID R01-R03 for whole-cell lysate samples (WCL) from the wild-type strain; data set ID R04-R06 for whole-cell lysate samples (WCL) from the triple mutant strain; data set ID R14-R16 for membrane fraction samples from the wild-type strain; and data set ID R17-R19 for membrane fraction samples (WCL) from the triple mutant strain. Interactive volcano plots of these data can be accessed at https://bit.ly/379SM6R (WCL) and https://bit.ly/35kHHNV (MEM).

**(ii) WGS.** Whole-genome sequences of the double and triple knockout strains were uploaded to the European Nucleotide Archive (ENA; https://www.ebi.ac.uk/) with the study number PRJEB40766.
