# Supplementary material for: An Unprecedented Tolerance to Deletion of the Periplasmic Chaperones SurA, Skp, and DegP in the Nosocomial Pathogen Acinetobacter baumannii
Source: J Bacteriol. 2022 Sep 15;204(10):e00054-22. doi: 10.1128/jb.00054-22 (PMC9578438; doi:10.1128/jb.00054-22)
Supplement: Supplemental file 1 — Fig. S1 to S6 and Tables S1 to S6. Download jb.00054-22-s0001.pdf, PDF file, 7.6 MB [file jb.00054-22-s0001.pdf]

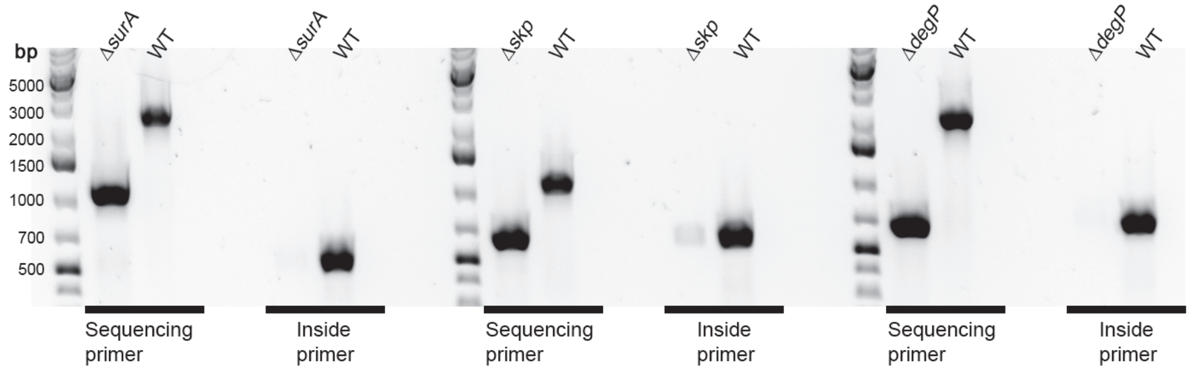

Figure S1 PCR verification of single knockout mutants. Deletion of the target genes was verified by PCRs using genomic DNA of the mutants as a template. One PCR was carried out using the same primer pair as used for sequencing of the target region after mutagenesis. These primers anneal to the flanking regions of the gene and result in a larger PCR product for the wild type (WT) than the mutant in case of successful deletion. Additionally, "nested primers" were used. These primers can only anneal to the unmodified wild type gene, resulting in a PCR product only for the wild type. For a list of primers used and the expected size of the PCR products please refer to **Table S1**.

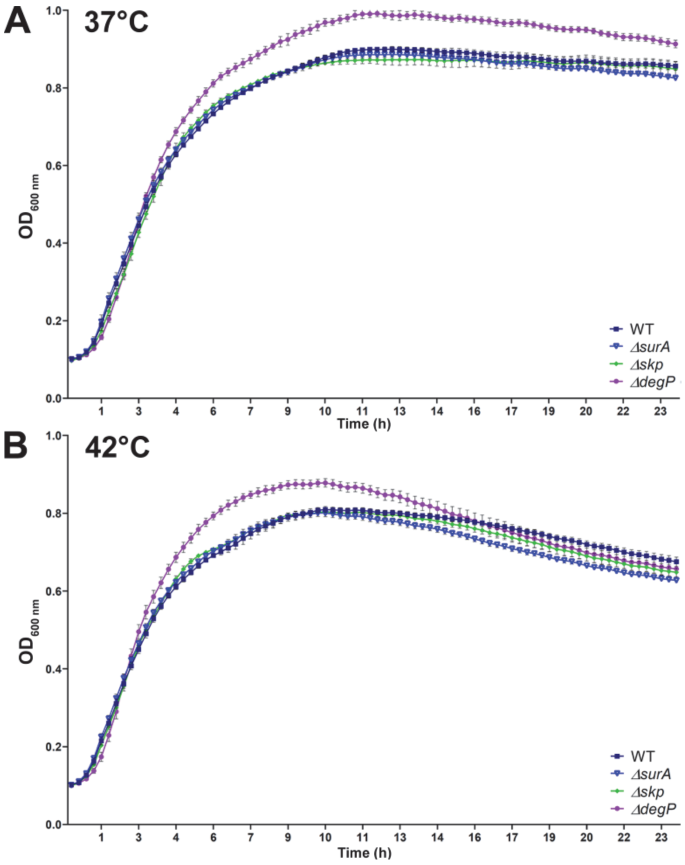

Figure S2 Effect of single chaperone gene deletions on the growth of AB5075. (A) To monitor the growth behavior of the indicated single null mutant strains in comparison to the wild type strain (WT), growth curves were recorded in quadruplicate and 3 independent experiments. The initial inoculum was adjusted to 1 x 10^7 cells per ml and cells were grown with shaking at 37°C or **(B)** 42°C in a 24-well plate.

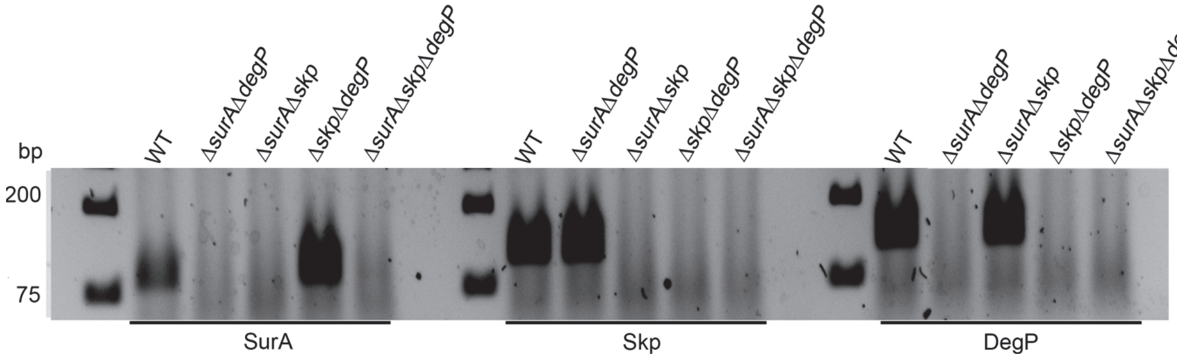

Figure S3 Qualitative RT-PCR validation of double and triple knockout strains. To check for the absence of mRNA of the genes that were knocked out qualitative RT-PCR was carried out. Total RNA was isolated from the wild type (WT), the double and triple knockout strains as indicated, reverse transcribed and amplified using primer pairs for *surA*, *skp* and *degP*. The primers used are listed in **Table S4**. A stained agarose gel of PCR products is shown.

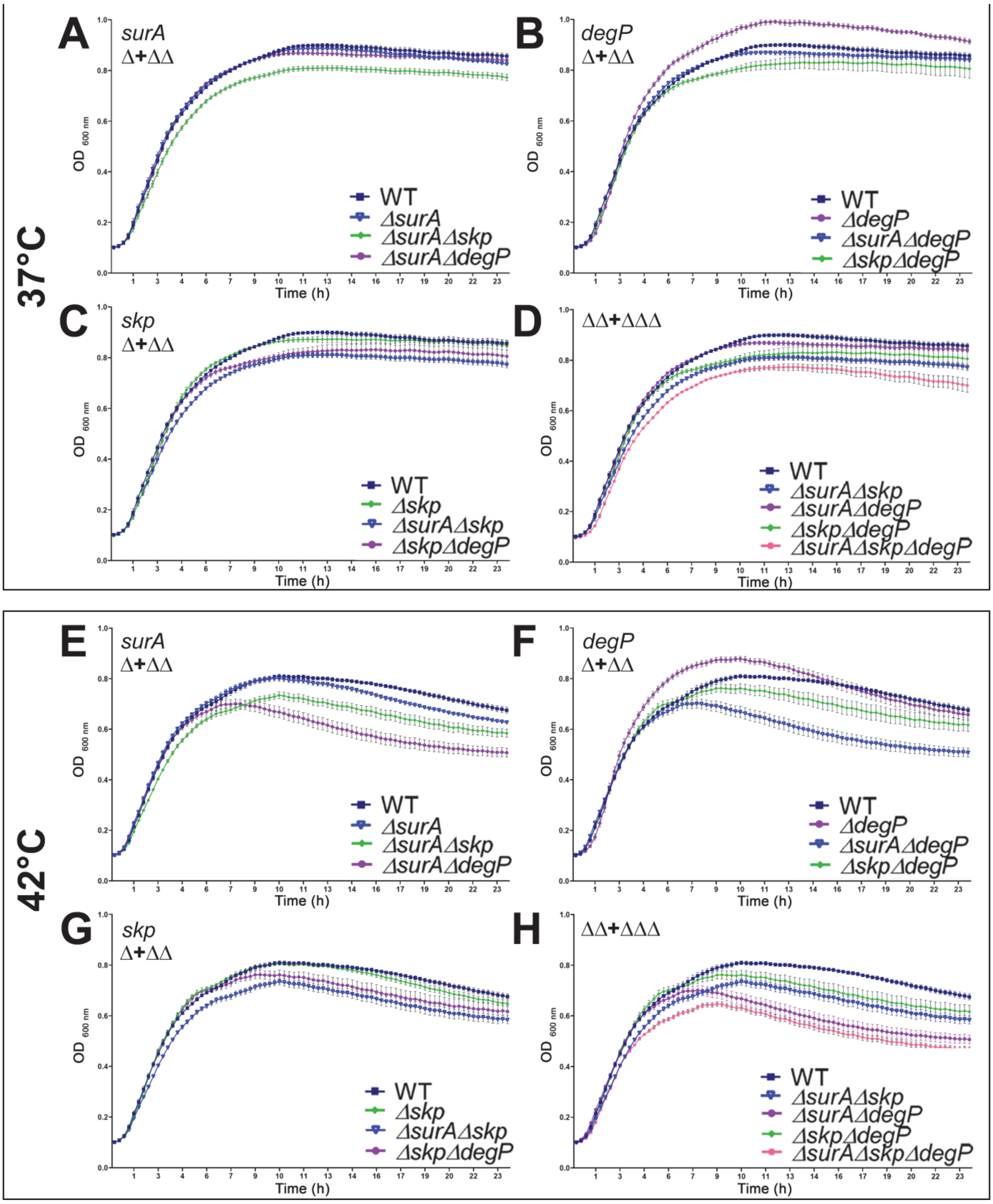

Figure S4 Effect of double and triple chaperone gene deletions on the growth of AB5075. To monitor the growth behavior of the double and triple mutant strains in comparison to the wild type (WT) strain, growth curves were recorded in quadruplicate and three independent experiments. The initial inoculum was adjusted to 1 x 10^7 cells per ml and cells were grown with shaking at (**A-D**) 37°C or (**E-H**) 42°C in a 24-well plate.

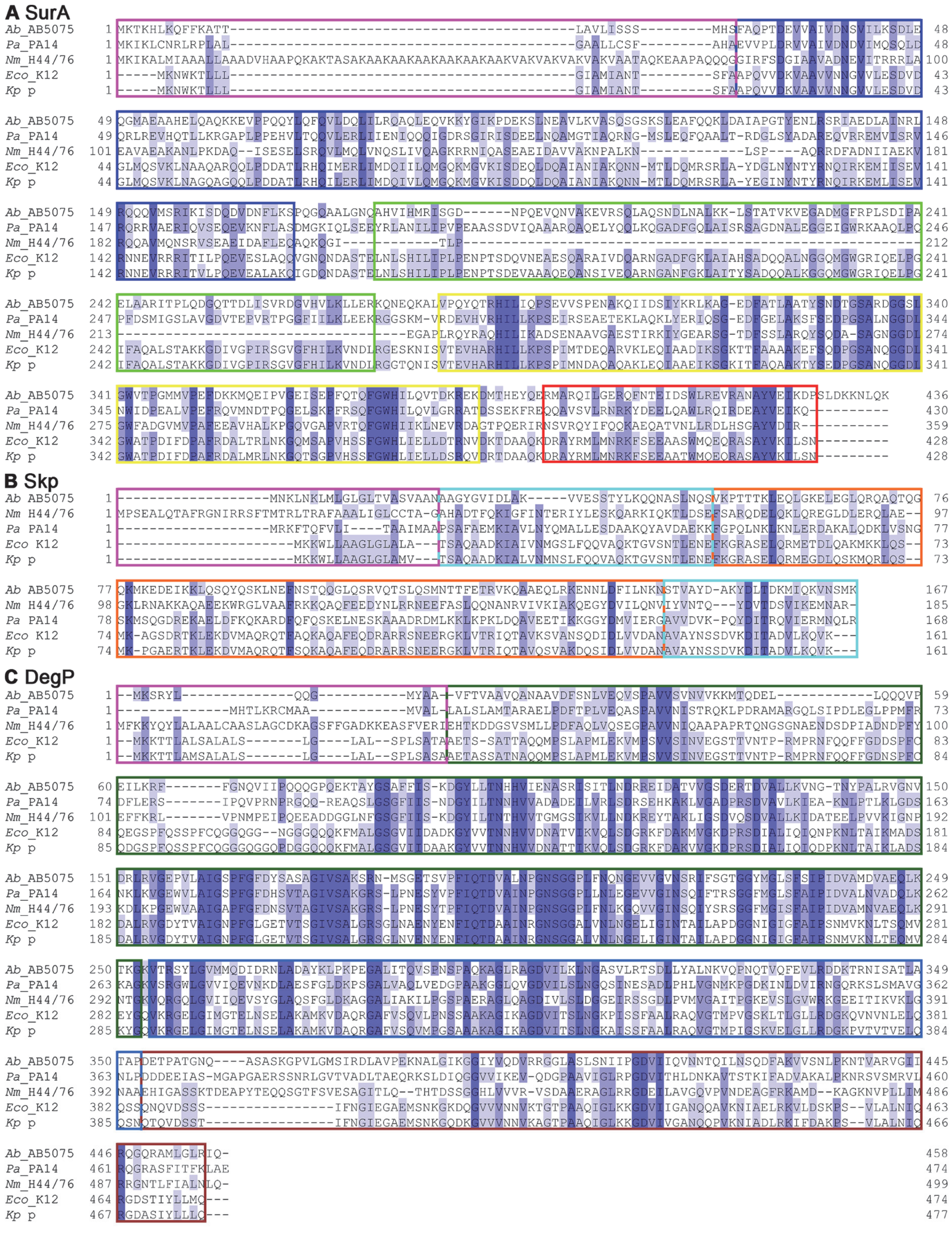

Figure S5 Amino acid sequence alignment and sequence conservation analysis of SurA, Skp and DegP of various Gram-negative species. **(A)** Sequence alignment of SurA from *Acinetobacter baumannii* strain AB5075 (*Ab* AB5075; A0A077GP18), *Pseudomonas aeruginosa* strain PA14 (*Pa* PA14*;* A0A0H2ZLB3), *Neisseria meningitidis* strain H44/76 (*Nm* H44/76; E6MWH2), *Klebsiella pneumoniae subsp. pneumoniae* (*Kp* p*;* A0A2X3H098) and

Escherichia coli strain K12 (*Eco* K12*;* P0ABZ6). Sequences were aligned using clustalΩ (1, 2) and color coded according to conservation from white (no conservation) to blue (100% conservation). Sequence identities for the full length proteins as well as the individual domains are summarized in **Table S3**. The domains are deduced from the crystal structure of the *Eco* K12 protein (pdb ID: 1m5y) and are indicated as a **magenta** box for the N-terminal signal sequence, as a **dark blue** box for the N-terminal domain, as a **green** box for the peptidyl-prolyl isomerase (PPI) domain 1, as a **yellow** box for the PPI2 domain and as a **red** box for the C-terminal domain. Of note, the N-terminal and C-terminal domains form a single structural domain, called the NC-core. The sequence alignment view was prepared with Jalview (3). (**B)** Sequence alignment of Skp as in A (*Ab* AB5075: V5VL3; *Nm* H44/76: E6MUY6; *Pa* PA14: A0A0H2ZEQ6; *Eco* K12: P0AEU7; *Kp* p: A0A1Y0Q2G3). The domains were deduced from the crystal structure of the *Eco* K12 protein (pdb IDs: 1sg2, 1u2m) and are indicated as **magenta** box for the N-terminal signal sequence, as **cyan** box for the body domain and as **orange** box for the tentacle domain of Skp. (**C)** Sequence alignment of DegP as in A (*Ab* AB5075: V5VAG2; *Pa* PA14: A0A0H2Z7B2; *Nm* H44/76: E6MVM8; *Eco* K12 P0C0V0; *Kp* p: A0A422Y8P3). The domains are deduced from the crystal structure of the *Eco* K12 protein (pdb IDs: 1ky9) and are indicated as a **magenta** box for the N-terminal signal sequence, as a **dark green** box for the DegP protease domain, as a **blue** box for the Postsynaptic density protein-95, Disk large, Zonula occludens 1 (PDZ) domain 1 and as a **brown** box for the PDZ2 domain.

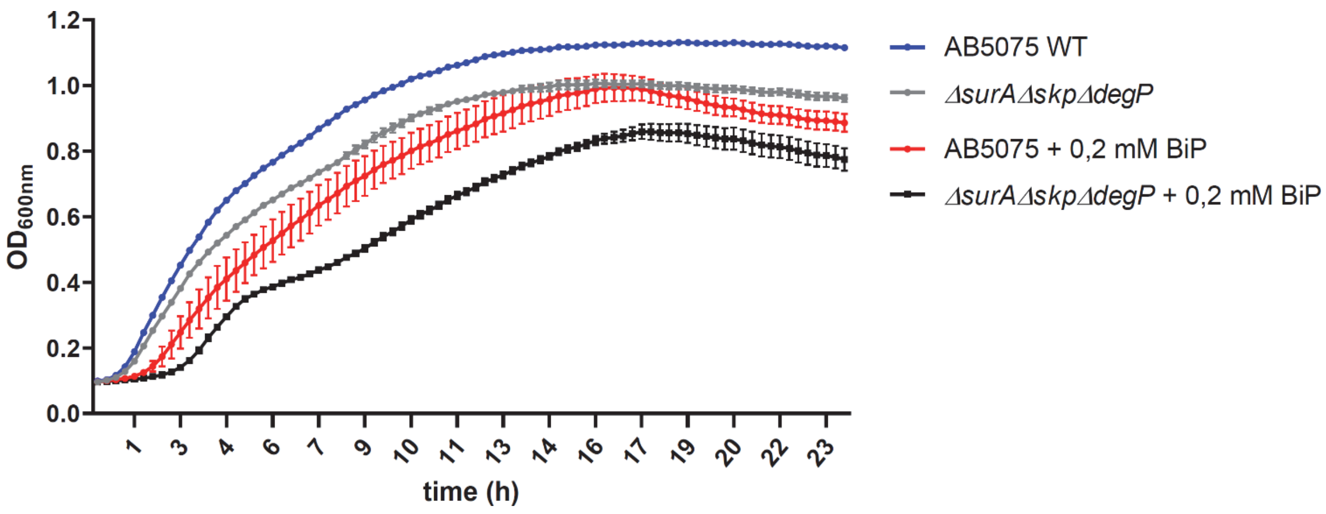

Figure S6 Growth behavior of AB5075 WT and the Δ*surA*Δ*skp*Δ*degP* triple mutant strain under iron replete and iron limiting (+ 0,2 mM BiP) conditions. Bacteria were inoculated into fresh LB medium with or without 0,2 mM 2,2′-Bipyridyl (BiP) and grown with shaking for 24 h at 37°C. The $OD_{600\ nm}$ was recorded every 15 min. Shown is one representative experiments with at least 3 technical replicates.

Table S1 Plasmids and strains used and generated in this study. The parent strain we used was Ab AB5075-UW (GeneBank assembly accession GCF_000963815.1).

**Plasmid**	**Resistance marker**	**Cassette for counter selection**	**Parent plasmid**	**Strains generated with**	**Source of plasmid**
**pVT77**	tellurite	*lacI*$_q$-*P*$_{trc}$-*lacO-tdk*	-	-	GenScript /(4)
***surA*_pVT77**	tellurite	*lacI*$_q$-*P*$_{trc}$-*lacO-tdk*	pVT77	AB5075_Δ*surA*<br>AB5075_ Δ*surA* / Δ*degP*<br>AB5075_ Δ*surA* / Δ*skp*<br>AB5075_Δ*surA*/Δ*skp*/Δ*degP*	GenScript
***skp*_pVT77**	tellurite	*lacI*$_q$-*P*$_{trc}$-*lacO-tdk*	pVT77	AB5075_Δ*skp*<br>AB5075_Δ*surA*/ Δ*skp*<br>AB5075_Δ*skp*/ Δ*degP*<br>AB5075_Δ*surA*/Δ*skp*/Δ*degP*	This study
***degP*_pVT77**	tellurite	*lacI*$_q$-*P*$_{trc}$-*lacO-tdk*	pVT77	AB5075_Δ*degP*<br>AB5075_Δ*surA*/ Δ*degP*<br>AB5075_Δ*skp*/ Δ*degP*<br>AB5075_Δ*surA*/Δ*skp*/Δ*degP*	This study

Table S2 Oligonucleotides used in this study

Gene	Name	Sequence (5´-3´)	Expected size of PCR product (bp)
**Oligonucleotides used for amplification of up- and downstream flanking regions of target genes**			
skp	skp_Up_fwd	AGTCAACGGCTGATATCCATTGCTGTTGACC GAAGCTGAATGATGACTATAAC	1165
	skp_Up_rev	TTATTTCATTGAATTAACCTTTTGTATCATCC CTAACATTAATTTATTTAATTTATTCATGT	
	skp_Down_fwd	ATGAATAAATTAAATAAATTAATGTTAGGG ATGATACAAAAGGTTAATTCAATGAAATAA A	1178
	skp_Down_rev	GGATCCCCGGGTACCGAGCTCGAATTCCTC CGGTCATAATATTCTATTTACGCAAA	
degP	degP_Up_fwd	ATTCGGAACCCAGCATGATATTCCGGAAAT ACAAGAAAGAACGTGCTTGCGGATG	1077
	degP_Up_rev	CATTTTTATAATATTTTACTGAATACGTAAA TAGCGAGATTTCATTCGGTGCTCACC	
	degP_Down_fwd	GTGGGTGAGCACCGAATGAAATCTCGCTAT TTACGTATTCAGTAAAATATTATAAAAATG AACC	1072
	degP_Down_rev	CCGCAAGCTTCCTGCAGGCTCTAGAGGATC AGTTAAGCTGATATTCCTGCCCG	
**Oligonucleotides used for sequencing of target region after knockout**			
surA	seq_ΔSurA_FWD	ATTGCTGCCAACTCGGCCTG	WT: 2363 Mutant: 1082
	seq_ΔSurA_REV	CAATTGCTGTAGGTACTTCACACG	
skp	seq_ΔSkp_fwd	GGTGACTGGACTCGACAAG	WT: 1061 Mutant: 617
	seq_ΔSkp_rev	GCTGCGGTAACAATGTAAGC	
degP	seq_ΔDegP_fwd	AACACCACCTTGTGCATAGAAG	WT: 1989 Mutant: 642
	seq_ΔDegP_rev	GCATCCACTGGACATACATTAC	

**Oligonucleotides used for verification of deletion (inside primers; bind in the region intended to be deleted)**			
surA	Validation_ΔSurA_negative_fwd	CAAACGATACAGGTTCAGCAC	WT: 515<br>Mutant: -
	Validation_ΔSurA_rev	GTACAGCTGGTAATGCTGACAAG	
skp	Validation_ΔSkp_negative_fwd	CAGCAAGGCTTACAGTCAAGAG	WT: 607<br>Mutant: -
	Validation_ΔSkp_rev	CGCCAACTACACAATTCTCAC	
degP	Validation_ΔDegP_negative_fwd	CGGTAATCAAGCCTCTGCTTC	WT: 626<br>Mutant: -
	Validation_ΔDegP_rev	CTTTACGGTATTGCACATGGTG	
**Oligonucleotides used for the detection of specific gene transcripts by RT-PCR**			
surA	RT-PCR_ΔSurA_FWD	TCCGAAGTGGTAAGCCCTGA	82
	RT-PCR_ΔSurA_REV	TAGCGAAATCTTCACCCGCT	
skp	RT-PCR_ΔSkp_FWD	ACGTCAAGCGCAGACTCAA	113
	RT-PCR_ΔSkp_REV	ACTGTAAGCCTTGCTGTGTTG	
degP	RT-PCR_ΔDegP_FWD	CCACAGCAACAAGGTCCTCA	116
	RT-PCR_ΔDegP_REV	ATACTGATGCGGGAAGCGTT	

Table S3 Pairwise identities (%) of SurA, Skp and DegP from various species. Domain nomenclature as in Fig. S5.

Protein	SurA Full length *Ab* AB5075	*Pa* PA14	*Kp* p	*Nm* H44/76	*Eco* K12	Skp Full length *Ab* AB5075	*Pa* PA14	*Kp* p	*Nm* H44/76	*Eco* K12	DegP Full length *Ab* AB5075	*Pa* PA14	*Kp* p	*Nm* H44/76	*Eco* K12
Ab AB5075		35,57	27,67	24,68	27,44		20,83	18,79	16,27	18,79		41,35	32,92	38,48	33,05
Pa PA14			39,02	26,75	38,41			23,49	22,16	25,15			38,65	47,07	39,05
Kp p				21,98	89,49				25,00	90,06				34,21	90,78
Nm H44/76					23,15					23,17					32,19
Eco K12															
Domain	NC-core					Body					Protease				
Ab AB5075		37,64	29,61	22,60	29,61		21,15	14,00	19,61	12,00		50,00	36,88	47,83	37,16
Pa PA14			35,80	22,86	36,36			29,09	30,36	27,27			42,75	56,52	43,68
Kp p				20,57	93,22				21,82	90,91				38,93	93,87
Nm H44/76					20,00					16,36					37,45
Eco K12															
Domain	PPI1					Tentacle					PDZ1				
Ab AB5075		26,88	19,79	n/a	19,15		20,21	20,83	14,13	18,95		36,36	34,34	36,36	35,35
Pa PA14			41,41	n/a	41,41			22,34	17,02	22,34			37,37	48,48	37,37
Kp p				n/a	87,88				23,91	86,81				37,37	87,88
Nm H44/76					n/a					20,65					34,34
Eco K12															
Domain	PPI2					-					PDZ2				
Ab AB5075		45,19	37,14	38,46	36,19							27,88	25,00	25,96	26,09
Pa PA14			42,86	34,62	41,90								30,93	29,36	31,96
Kp p				37,14	85,71									25,56	84,44
Nm H44/76					38,10										22,22
Eco K12															

Table S4 Search for SurA, Skp and DegP paralogues in AB5075. To identify paralogues of the AB5075 proteins SurA, Skp and DegP the AB5075 proteome (UniProt ID UP000032746) (5) was scanned for sequences homologous to SurA (A0A077GP18), Skp (V5VCL3) and DegP (V5VAG2) using BLAST (2.10.0+) (6). BLAST hits of the query sequences are highlighted in italic. Sequence alignments identified by BLAST were manually inspected and the homologous regions were assigned to the corresponding regions of the query proteins (**Fig. S5**). In addition, their (predicted) localization was retrieved from the InterPro database (7). Finally, BLAST hits were qualified as highly probable (green) depending on whether all three criteria were met and less probable (yellow) paralogues if at least one criterion was not met. First, they must have a sequence identity >= 30% and an as high as possible percentage of positives. Secondly, the sequence identity must be in a substantial sequence range (>=60 aa). Third, they must be (predicted to) localize to the periplasm. However, the search for paralogues by simple sequence homology search algorithms such as BLAST does not allow identifying functional paralogues with no or limited sequence homology. Therefore, the existence of additional paralogues cannot be ruled out.

accession number	name	score (Bits)	E value	localisation	size (aa)	% identity	% positives	domain homology	possible orthologes	comments
**SurA**										
A0A077GP18	*Chaperone SurA*	*893*	*0*	*periplasm*	*436*	*100*			*n/a*	
V5VA69	Peptidylprolyl isomerase PpiC	60,5	2,00E-12	cytoplasm	96	37	51	93 aa, PPI2	no	PPI2 domain
A0A0E1JKW1	Peptidylprolyl isomerase PpiD	65,1	3,00E-12	inner membrane transmembrane protein, mostly periplasmic	621	35	61	110 aa, PPI2	yes	PPI2 domain
A0A0D5YFT8	Uncharacterized protein	25,8	2,4	cytoplasm	110	24	40	86 aa, N	no	
A0A0D5YFC4	Patatin-like phospholipase family protein	26,6	3,6	lipoprotein	305	28	47	79 aa, PPI1	maybe	
A0A0D5YCP6	Cation efflux system protein (EsvF1)	26,2	4,6	inner membrane transmembrane protein, mostly periplasmic	405	52	71	21 aa, N	no	
A0A0D5YG98	AMP-binding protein	25,8	5,8	cytoplasm	547	36	50	28 aa, N	no	
A0A0D5YHN3	Transaldolase	25,4	6,7	cytoplasm	329	27	50	45 aa, PPI1	no	
A0A0D5YJX4	3-oxoadipyl-CoA thiolase	25,4	8,2	inner membrane protein, mostly cytoplasmic	401	28	52	87 aa, N	no	
A0A0D5YK63	Capsule assembly Wzi family protein	25,4	8,7	outer membrane protein	481	28	52	46 aa, C	no	
A0A0D5YFQ2	Rhombotarget A	25,4	9	outer membrane protein	617	31	51	39 aa, N-PPI1	no	
**Skp**										
V5VCL3	*OmpH family outer membrane protein Skp*	*331*	*2,00E-119*	*periplasm*	*167*	*100*			*n/a*	
A0A0D5YDF1	NAD-dependent succinate-semialdehyde dehydrogenase	26,9	0,6	cytoplasm	482	27	50	52 aa, tentacle	no	
A0A0D5YKR2	Uncharacterized protein	25	1,7	?	136	25	48	60 aa, tentacle	no	
A0A077GFJ9	4-hydroxybenzoate octaprenyltransferase	25,4	1,8	inner membrane transmembrane protein	292	30	45	47 aa, tentacle	no	
V5VAP2	ABC transporter ATP-binding protein	25,4	2,1	inner membrane transmembrane protein	553	36	64	44 aa, body-tentacle	no	
A0A0D5YIG3	Bifunctional (P)ppGpp synthase/hydrolase SpoT	24,6	2,5		146	29	50	84 aa, tentacle	no	
V5VI16	Arginine--tRNA ligase	25	2,9	cytoplasm	596	29	53	34 aa, tentacle	no	
A0A0J8W8F3	Heat shock protein HtpX	23,5	8,1	inner membrane transmembrane protein	629	34	62	29 aa, tentacle	no	
**DegP**										
V5VAG2	*Periplasmic serine endoprotease DegP-like*	*919*	*0*	*periplasm*	*458*	*100*			*n/a*	
A0A0D5YGS6	Peptidase S1 and S6/ DegS	187	8,00E-56	inner membrane transmembrane protein, mostly periplasmic	391	39	57	293 aa, protease-PDZ1	yes	protease and PDZ1 domain
A0A0D5YFR0	M61 family peptidase	36,6	0,003	cytoplasm	567	29	45	139 aa, protease-PDZ1	no	
A0A086HVC9	General secretion pathway protein	34,7	0,008	inner membrane transmembrane protein, mostly periplasmic	278	32	52	65 aa, PDZ1	maybe	PDZ1 domain
A0A0D5YI62	Zinc metalloprotease	33,1	0,033	inner membrane transmembrane protein, mostly periplasmic	451	34	51	61 aa, PDZ1	maybe	PDZ1 domain, contains 2 PDZ domains
V5VCS2	Ribose-5-phosphate isomerase A	28,9	0,57	cytoplasm	223	46	69	26 aa, PDZ2	no	
G1D8S2	APH(3')-VI family aminoglycoside O-phosphotransferase	26,2	4	cytoplasm	259	28	53	47 aa, protease	no	
A0A0D5YKM0	Endonuclease/exonuclease/phosphatase	26,2	4,8	cytoplasm	783	26	42	95 aa, protease	no	
A0A0D5YJ06	Urea amidolyase	26,2	5,7	cytoplasm	1201	42	67	24 aa, PDZ2	no	
V5VI16	Arginine--tRNA ligase	25,4	8	cytoplasm	596	33	21	43 aa, PDZ1-PDZ2	no	

Table S5 Copy number variation analysis for A0A0D5YIP3 and A0A0D5YJ02. Genes for the two proteins were extracted from Uniprot and a BLASTn search was performed for each gene independently. The BLASTn search was restricted to *Acinetobacter baumannii*. The resulting hits were checked for overlaps and summerized for each reference strain over all genes. The table indicates the GenBank IDs of the strain genomes (Reference), strain names (strain) and the number of copies for A0A0D5YIP3 and A0A0D5YJ02.

Reference	Strain	Copies A0A0D5YIP3	Copies A0A0D5YJ02
CP001182.2	Acinetobacter baumannii AB0057	0	6
CP012006.1	Acinetobacter baumannii Ab04-mff	0	1
AP022077.1	Acinetobacter baumannii DNA, complete genome, strain: WP4-W18-ESBL-11	1	2
CP007712.1	Acinetobacter baumannii LAC-4	0	1
AP023077.1	Acinetobacter baumannii OCU_Ac16a DNA	0	2
CP041035.1	Acinetobacter baumannii strain 11W359501	0	5
CP044517.1	Acinetobacter baumannii strain 31FS3-2	2	0
CP045541.1	Acinetobacter baumannii strain 5457	0	5
CP022283.1	Acinetobacter baumannii strain 7804	1	0
CP024418.1	Acinetobacter baumannii strain A388	0	4
CP021782.1	Acinetobacter baumannii strain A85	0	3
CP037872.1	Acinetobacter baumannii strain AB046	1	0
CP091367.1	Acinetobacter baumannii strain AB169-VUB	2	2
CP091340.1	Acinetobacter baumannii strain AB227-VUB	0	1
CP091339.1	Acinetobacter baumannii strain AB229-VUB	0	1
CP091338.1	Acinetobacter baumannii strain AB231-VUB	2	0
CP091337.1	Acinetobacter baumannii strain AB232-VUB	0	1
CP083181.1	Acinetobacter baumannii strain AB43	1	2
**CP008706.1**	**Acinetobacter baumannii strain AB5075-UW**	**3**	**6**
CP070362.2	Acinetobacter baumannii strain AB5075-VUB	3	7
CP070358.2	Acinetobacter baumannii strain AB5075-VUB-itrA::ISAba13	5	9
CP045428.1	Acinetobacter baumannii strain AbCAN2	1	0
KM998768.1	Acinetobacter baumannii strain AO-21841 CRISPR-cas3/cas2 array genomic sequence	0	1
CP027183.1	Acinetobacter baumannii strain AR_0052	1	0
CP027178.1	Acinetobacter baumannii strain AR_0070	1	0
CP081137.1	Acinetobacter baumannii strain ARLG_6420	1	0
CP058625.1	Acinetobacter baumannii strain ATCC BAA1605	0	5
CP021342.1	Acinetobacter baumannii strain B8342	0	2
CP060994.1	Acinetobacter baumannii strain CAb-65	0	2
CP044356.1	Acinetobacter baumannii strain CAM180-1	1	0
CP038500.1	Acinetobacter baumannii strain CIAT758	0	1
CP012952.1	Acinetobacter baumannii strain D36	0	4
CP030106.1	Acinetobacter baumannii strain DA33382	0	2
CP027704.2	Acinetobacter baumannii strain DS002	0	1
CP091333.1	Acinetobacter baumannii strain DSM30011-VUB	0	3
CP066016.1	Acinetobacter baumannii strain FDAARGOS_1036	0	5
CP033754.1	Acinetobacter baumannii strain FDAARGOS_540	0	2
CP018677.1	Acinetobacter baumannii strain LAC4	0	2
LS483472.1	Acinetobacter baumannii strain NCTC13421 genome assembly, chromosome: 1	0	5
CP043180.1	Acinetobacter baumannii strain PG20180064	1	0
CP050432.1	Acinetobacter baumannii strain PM194229	0	2
CP064292.1	Acinetobacter baumannii strain SD	2	0
CP087594.1	Acinetobacter baumannii strain SHOU-Ab01	2	0
CP040080.1	Acinetobacter baumannii strain SP304_c	1	0
CP020595.1	Acinetobacter baumannii strain USA15	0	3
CP050403.1	Acinetobacter baumannii strain VB2486	0	2
CP027246.2	Acinetobacter baumannii strain WCHAB005078	0	2
CP020598.1	Acinetobacter baumannii strain WKA02	0	2
CP054560.1	Acinetobacter baumannii strain YC103	2	0

Table S6 SNP analysis. Using the assemblies generated from the WGS of our AB5075 WT lab strain and mutant strains, we performed a SNP analysis. The tool Snippy (41) was used with the default settings and as a reference the *Acinetobacter baumannii* strain AB5075-UW was used. Type of SNPs (snp = single nucleotide polymorphism, del = deletion, ins = insertion or complex), and details of the SNPs are indicated.

TYPE	REF	ALT	EVIDENCE	FTYPE	STRAND	AA_POS	EFFECT	GENE	PRODUCT	Gene ID	
snp	T	C	C:20 T:0	CDS	+	199/1148	synonymous_variant c.597T>C p.Gly199Gly		host specificity factor TipJ family phage tail protein	ABUW_0808	5075 WT Ref vs. ΔΔΔ
snp	A	T	T:20 A:0	CDS	+	245/1148	synonymous_variant c.735A>T p.Ala245Ala		host specificity factor TipJ family phage tail protein	ABUW_0808	
snp	G	A	A:20 G:0	CDS	+	248/1148	synonymous_variant c.744G>A p.Gln248Gln		host specificity factor TipJ family phage tail protein	ABUW_0808	
snp	C	T	T:20 C:0	CDS	+	264/1148	synonymous_variant c.792C>T p.Ile264Ile		host specificity factor TipJ family phage tail protein	ABUW_0808	
snp	G	T	T:20 G:0	CDS	+	56/388	missense_variant c.167G>T p.Gly56Val	metK	methionine adenosyltransferase	ABUW_2296	
snp	A	C	C:20 A:0	CDS	-	648/899	synonymous_variant c.1944T>G p.Gly648Gly	infB	translation initiation factor IF-2	ABUW_3541	
snp	C	A	A:20 C:0	CDS	+	316/337	missense_variant c.947C>A p.Ala316Asp	hemB	porphobilinogen synthase	ABUW_3022	
snp	C	T	T:20 C:0	CDS	+	318/337	missense_variant c.953C>T p.Ala318Val	hemB	porphobilinogen synthase	ABUW_3022	
snp	C	T	T:20 C:0	CDS	-	227/356	missense_variant c.679G>A p.Glu227Lys		ABC transporter permease	ABUW_0966	5075 WT Ref vs. Δskp ΔdegP
snp	T	G	G:20 T:0	CDS	+	176/404	synonymous_variant c.528T>G p.Gly176Gly		MFS transporter	ABUW_2683	
snp	C	A	A:20 C:0	CDS	-	677/949	missense_variant c.2029G>T p.Gly677Cys		EAL domain-containing protein	ABUW_1221	5075 WT Ref vs. ΔsurAΔdegP
snp	T	G	G:20 T:0	CDS	-	153/476	missense_variant c.459A>C p.Lys153Asn		ABC transporter substrate-binding protein	ABUW_2111	
snp	T	A	A:20 T:0	CDS	+	109/240	synonymous_variant c.327T>A p.Gly109Gly		AzlC family ABC transporter permease	ABUW_2347	
snp	C	A	A:20 C:0	CDS	+	103/240	missense_variant c.308C>A p.Thr103Lys		AzlC family ABC transporter permease	ABUW_2347	
snp	C	T	T:20 C:0	CDS	+	353/496	stop_gained c.1057C>T p.Gln353*		NAD(P)/FAD-dependent oxidoreductase	ABUW_0433	5075 WT Ref vs. ΔsurAΔskp
snp	T	C	C:20 T:0	CDS	+	313/513	missense_variant c.938T>C p.Leu313Ser	purF	amidophosphoribosyltransferase	ABUW_1446	
snp	G	A	A:20 G:0	CDS	-	245/527	missense_variant c.733C>T p.Arg245Cys		urea amidolyase family protein	ABUW_2604	
del	AG	A	A:17 AG:0	tRNA	-		intragenic_variant n.3099210delG		tRNA-Met	ABUW_3071	
ins	G	GT	GT:17 G:0	tRNA	-		intragenic_variant n.3099214_3099215insT		tRNA-Met	ABUW_3071	
complex	CGCA	ACGC	ACGC:17 CGCA:0	tRNA	-		intragenic_variant n.3099200_3099203delCGCAinsACGC		tRNA-Met	ABUW_3071	
snp	G	A	A:20 G:0	CDS	+	207/218	synonymous_variant c.621G>A p.Gly207Gly		hypothetical protein	ABUW_1409	5075 WT Ref vs. Lab WT

Supplementary References

1. Sievers F, Wilm A, Dineen D, Gibson TJ, Karplus K, Li W, Lopez R, McWilliam H, Remmert M, Soding J, Thompson JD, Higgins DG. 2011. Fast, scalable generation of high-quality protein multiple sequence alignments using Clustal Omega. Mol Syst Biol 7:539.doi:10.1038/msb.2011.75.
2. Zimmermann L, Stephens A, Nam SZ, Rau D, Kubler J, Lozajic M, Gabler F, Soding J, Lupas AN, Alva V. 2018. A Completely Reimplemented MPI Bioinformatics Toolkit with a New HHpred Server at its Core. J Mol Biol 430:2237-2243.doi:10.1016/j.jmb.2017.12.007.
3. Waterhouse AM, Procter JB, Martin DM, Clamp M, Barton GJ. 2009. Jalview Version 2--a multiple sequence alignment editor and analysis workbench. Bioinformatics 25:1189-91.doi:10.1093/bioinformatics/btp033.
4. Trebosc V, Gartenmann S, Royet K, Manfredi P, Totzl M, Schellhorn B, Pieren M, Tigges M, Lociuro S, Sennhenn PC, Gitzinger M, Bumann D, Kemmer C. 2016. A Novel Genome-Editing Platform for Drug-Resistant *Acinetobacter baumannii* Reveals an AdeR-Unrelated Tigecycline Resistance Mechanism. Antimicrob Agents Chemother 60:7263-7271.doi:10.1128/AAC.01275-16.
5. Gallagher LA, Ramage E, Weiss EJ, Radey M, Hayden HS, Held KG, Huse HK, Zurawski DV, Brittnacher MJ, Manoil C. 2015. Resources for Genetic and Genomic Analysis of Emerging Pathogen *Acinetobacter baumannii*. J Bacteriol 197:2027-35.doi:10.1128/JB.00131-15.
6. Altschul SF, Madden TL, Schaffer AA, Zhang J, Zhang Z, Miller W, Lipman DJ. 1997. Gapped BLAST and PSI-BLAST: a new generation of protein database search programs. Nucleic Acids Res 25:3389-402.doi:10.1093/nar/25.17.3389.
7. Mitchell AL, Attwood TK, Babbitt PC, Blum M, Bork P, Bridge A, Brown SD, Chang HY, El-Gebali S, Fraser MI, Gough J, Haft DR, Huang H, Letunic I, Lopez R, Luciani A, Madeira F, Marchler-Bauer A, Mi H, Natale DA, Necci M, Nuka G, Orengo C, Pandurangan AP, Paysan-Lafosse T, Pesseat S, Potter SC, Qureshi MA, Rawlings ND, Redaschi N, Richardson LJ, Rivoire C, Salazar GA, Sangrador-Vegas A, Sigrist CJA, Sillitoe I, Sutton GG, Thanki N, Thomas PD, Tosatto SCE, Yong SY, Finn RD. 2019. InterPro in 2019: improving coverage, classification and access to protein sequence annotations. Nucleic Acids Res 47:D351-D360.doi:10.1093/nar/gky1100.
